# Placental dysfunction influences fetal monocyte subpopulation gene expression in preterm birth

**DOI:** 10.1172/jci.insight.155482

**Published:** 2022-06-08

**Authors:** Abhineet M. Sharma, Robert Birkett, Erika T. Lin, Linda M. Ernst, William A. Grobman, Suchitra Swaminathan, Hiam Abdala-Valencia, Alexander V. Misharin, Elizabeth T. Bartom, Karen K. Mestan

**Affiliations:** 1 Department of Pediatrics/Division of Neonatology, Northwestern University Feinberg School of Medicine, Chicago, Illinois, USA.; 2 Department of Pediatrics, UCSD, La Jolla, California, USA.; 3 Department of Pathology & Laboratory Medicine, NorthShore University HealthSystem, Chicago, Illinois, USA.; 4 Department of Obstetrics & Gynecology, Division of Maternal-Fetal Medicine,; 5 Department of Medicine/Division of Rheumatology,; 6 Department of Medicine/Division of Pulmonary & Critical Care, and; 7 Department of Biochemistry and Molecular Genetics, Northwestern University Feinberg School of Medicine, Chicago, Illinois, USA.

**Keywords:** Reproductive Biology, Vascular Biology, Adaptive immunity, Chemokines, Monocytes

## Abstract

The placenta is the primary organ for immune regulation, nutrient delivery, gas exchange, protection against environmental toxins, and physiologic perturbations during pregnancy. Placental inflammation and vascular dysfunction during pregnancy are associated with a growing list of prematurity-related complications. The goal of this study was to identify differences in gene expression profiles in fetal monocytes — cells that persist and differentiate postnatally — according to distinct placental histologic domains. Here, by using bulk RNA-Seq, we report that placental lesions are associated with gene expression changes in fetal monocyte subsets. Specifically, we found that fetal monocytes exposed to acute placental inflammation upregulate biological processes related to monocyte activation, monocyte chemotaxis, and platelet function, while monocytes exposed to maternal vascular malperfusion lesions downregulate these processes. Additionally, we show that intermediate monocytes might be a source of mitogens, such as *HBEGF, NRG1, and VEGFA,* implicated in different outcomes related to prematurity. This is the first study to our knowledge to show that placental lesions are associated with unique changes in fetal monocytes and monocyte subsets. As fetal monocytes persist and differentiate into various phagocytic cells following birth, our study may provide insight into morbidity related to prematurity and ultimately potential therapeutic targets.

## Introduction

The placenta provides the primary means for immune regulation between the mother and fetus, and it provides nutrient delivery, gas exchange, and protection against environmental toxins and physiologic perturbations during pregnancy. Underlying acute and chronic processes of placental dysfunction are important causes of adverse pregnancy outcome, and distinct placental histologic findings at birth are indicators of these pathologic processes. In full-term births, inflammatory lesions in the placenta often accompany clinical manifestations of newborn sepsis/infection, while in preterm births with placental inflammation, acute chorioamnionitis is often the cause of spontaneous preterm labor. Alternatively, maternal vascular malperfusion (MVM) lesions that develop in earlier stages of pregnancy may be accompanied by fetal growth restriction and vascular dysregulation in preterm infants. Both inflammatory and vascular dysfunctions of the placenta may have a profound impact on offspring, especially among infants born preterm.

Distinct placental lesions that indicate immune and vascular dysfunction during pregnancy are associated with a growing list of neonatal complications of prematurity (1–7). While many of these adverse outcomes are highly complex, multifactorial in nature, and heterogeneous in their clinical course, there is growing evidence that the placenta plays a pivotal role in programming the fetal immune system, which may impact downstream postnatal environmental exposure and early life events (8). Central to the “fetal origins hypothesis” is the potential for the placenta to modulate circulating immune progenitor cells during pregnancy and at the time of delivery. Fetal monocytes circulating between the placenta and the fetus are potential mediators of disease, as well as important markers for enhancing our understanding of the complex interplay between the placenta and preterm neonate (9–12). Monocytes, which have historically been defined into subtypes based on CD14 and CD16 cell surface markers, can differentiate into different tissue-resident macrophages based on previous exposure and, therefore, may play an essential role in placental-fetal crosstalk (13–16).

The main objective of this study was to identify differences in gene expression profiles in fetal monocytes and their subsets, including by the distinct placental histologic domains that are associated with certain outcomes of preterm birth. We first investigated how classical, intermediate, and nonclassical monocyte concentrations vary across the gestational age (GA) spectrum and relative to other immune cell types circulating at birth. We applied RNA-Seq to investigate differences in gene expression across 4 major domains of placental histology: acute inflammation (AI), chronic inflammation (CI), MVM, and fetal vascular malperfusion (FVM). Lastly, we sought to identify differentially expressed genes in the context of placental histology in order to elucidate the role of fetal monocytes in adverse neonatal outcomes in the setting of placental dysfunction.

## Results

### The neonatal cohort covered a broad range of perinatal, placental, and postnatal findings.

A total of 70 mother-infant dyads were enrolled in our study and had cord blood available for cell counts, nucleic acid extraction, and sequencing. Of these, 59 were preterm deliveries and 11 were term deliveries (Table 1, Table 2, Table 3, Table 4). There were statistically significant differences in the rate of postnatal steroid therapy, maternal group B streptococcus (GBS) status, and cesarean delivery (*P* < 0.05) (Table 1). Those born preterm had higher rates of premature rupture of membrane (PROM) (Table 2). As expected, when compared with term neonates, preterm neonates were born at lower GA, had lower Apgar scores (a standardized tool to assess a newborn’s condition immediately after birth) at 1 and 5 minutes, had lower birth weights (BWs) and had a higher rate of neonatal intensive care unit admission (*P* < 0.001; Table 3). In total, 61 placentas were available for gross and histopathology examination (Table 4). Placentas that were not available for analysis were from term or late preterm infants. MVM was present in 83% of preterm births and 9% of term births (*P* < 0.001). The second most common domain represented was AI, representing 32% of preterm and 18% of term births.

### Placental histopathology does not correlate with composition of immune cells in cord blood.

**Figure 1:**
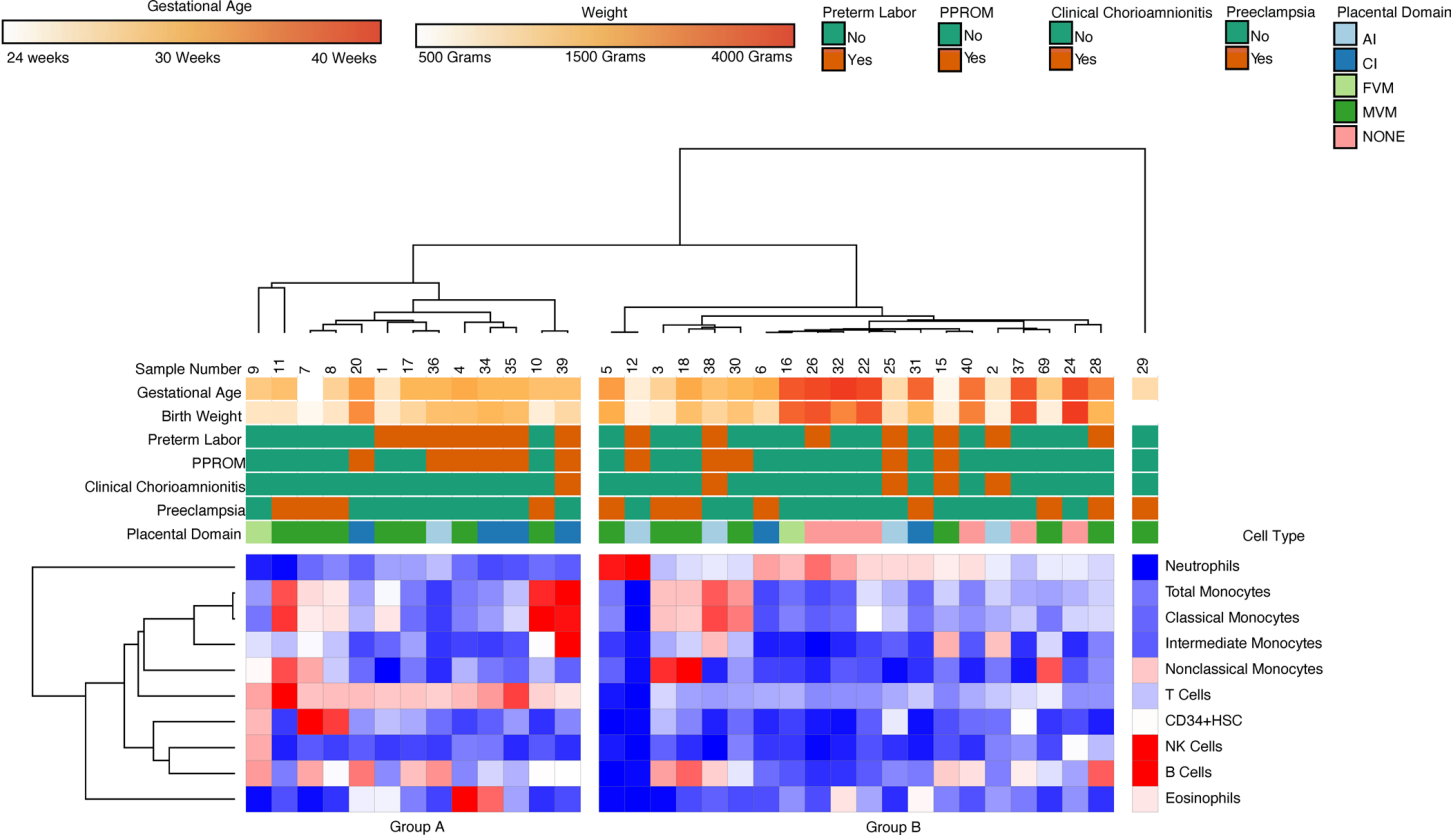
Hierarchical clustering on flow cytometry data revealed 2 major clusters. Group A was characterized by prevalence T cells and B cells, and Group B was characterized by increased abundance of neutrophils.

**Figure 2:**
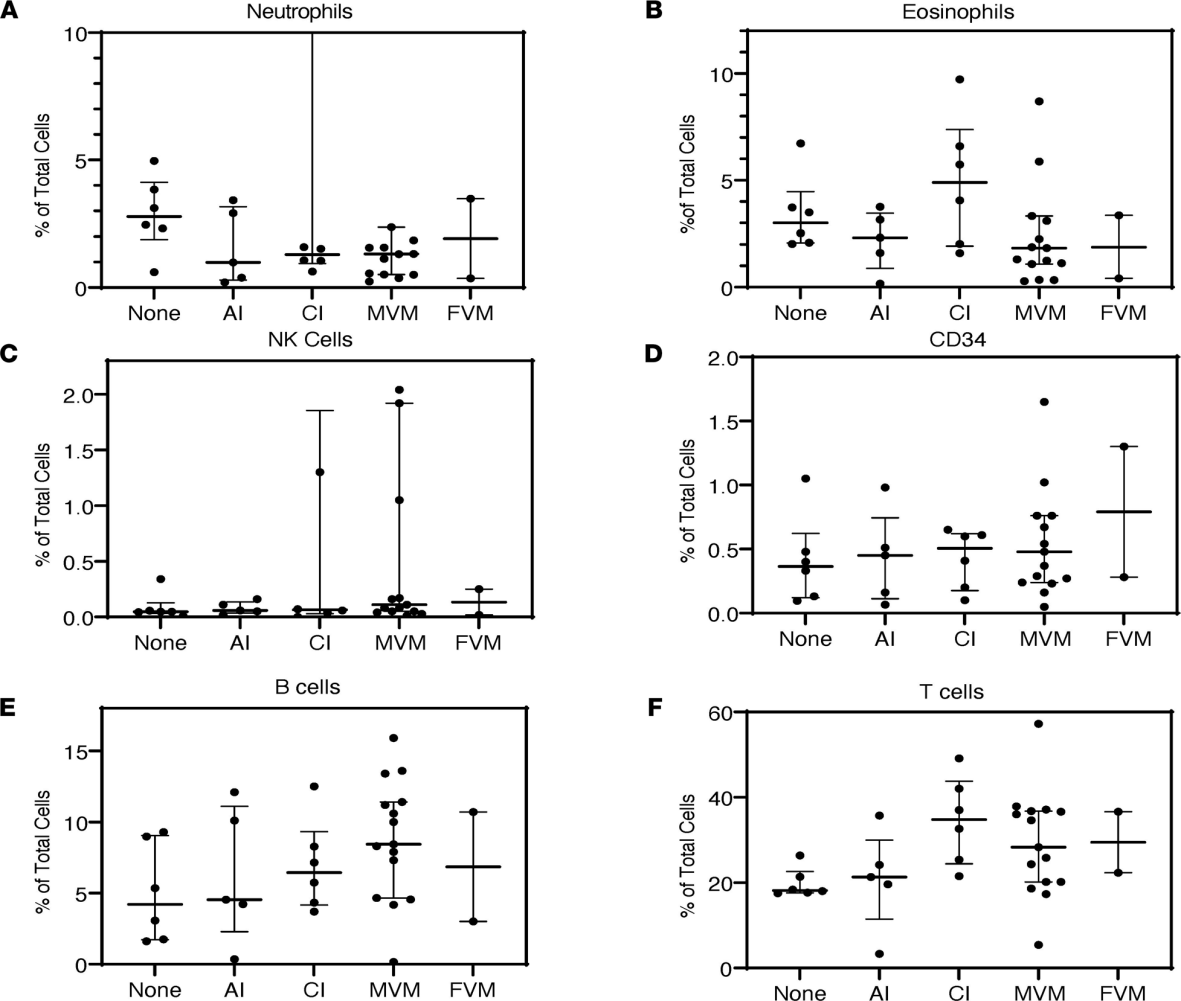
Cell composition by placental pathology domains. (**A**–**F**) There were no significant differences in cell composition when comparing neutrophils, eosinophils, NK Cells, CD34^+^, B cells, or T cells between different domains of placental pathology (none = 15 cases, AI = 13 cases, CI = 7 cases, MVM = 33 cases, FVM = 2 cases).

In order to characterize the relationship between placental pathology and infant cord blood composition, we performed flow cytometric profiling of cord blood collected immediately at birth (*n* = 34). Hierarchical clustering on flow cytometry data revealed 2 major clusters: a cluster (Group A) characterized by increased abundance of T cells and B cells and a cluster (Group B) characterized by prevalence of neutrophils (Figure 1). The clusters did not correlate with GA, maternal steroid therapy, fetal growth restriction, or primary placental domain. Furthermore, direct comparison of the cell type abundance between different domains of placental pathology did not identify significant differences (Figure 2).

### Placental histopathology is associated with changes in proportion of classical/intermediate and nonclassical monocyte populations.

**Figure 3:**
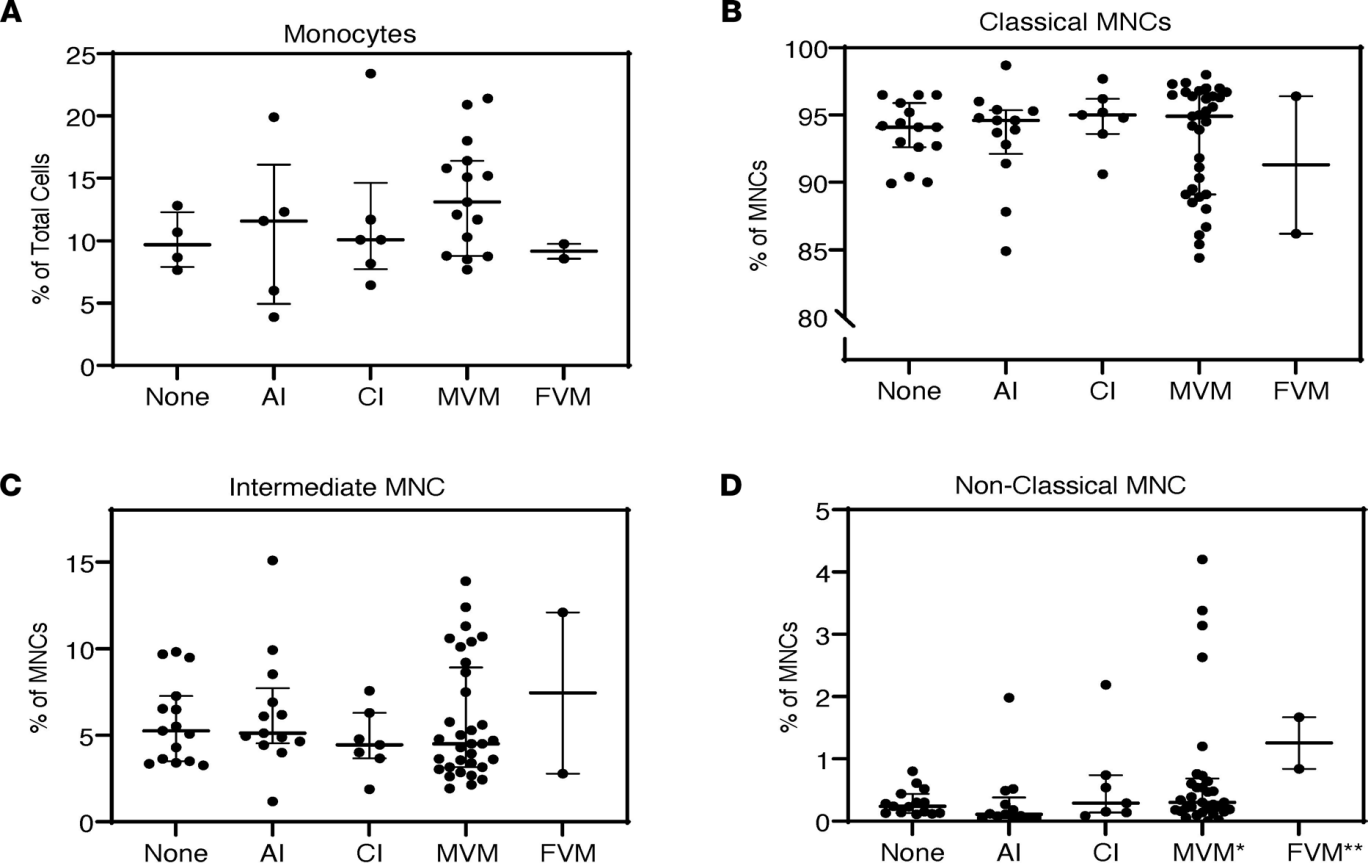
Monocyte subpopulations by placental pathology domains. (**A**–**D**) There were no significant differences in total population size when comparing different placental domains (none = 15 cases, AI = 13 cases, CI = 7 cases, MVM = 33 cases, FVM = 2 cases). For a total of 70 samples, we analyzed monocyte subtype composition and only found significant differences in the nonclassical subtype. These differences were between the AI to MVM (*P = 0.004), AI to FVM (**P = 0.005), and None to FVM (**P = 0.03) based on Kruskall-Wallis rank sum test.

Changes in differential abundance of monocyte subsets have been shown to be associated with cardiovascular events and vascular dysfunction in adults (17, 18). We therefore performed analysis of monocyte subsets (*n* = 70) between domains of placental pathology (none = 15 cases, AI = 13 cases, CI = 7 cases, MVM = 33 cases, FVM = 2 cases). The median (IQR) percentage of monocytes according to the 3 subpopulations were 94.6% (90.6%, 96.3%) for classical, 4.8% (3.6%, 7.5%) for intermediate, and 0.27% (0.13%, 0.54%) for nonclassical. Relative abundance of classical and intermediate monocytes was not different between primary domains of placental pathology (Figure 3). In contrast, percentage of nonclassical monocytes was significantly different according to placental domain (*P* < 0.05). The median percentage of nonclassical MNCs was significantly higher in the MVM and FVM groups as compared with infants with AI placentas (Figure 3). To investigate whether different placental domains manifested in differential activation of monocytes, we applied a more sensitive technology — gene expression profiling (RNA-Seq).

### Transcriptomic profiling of cord blood monocyte subsets identifies cell type–specific changes associated with different domains of placental pathology.

**Figure 4:**
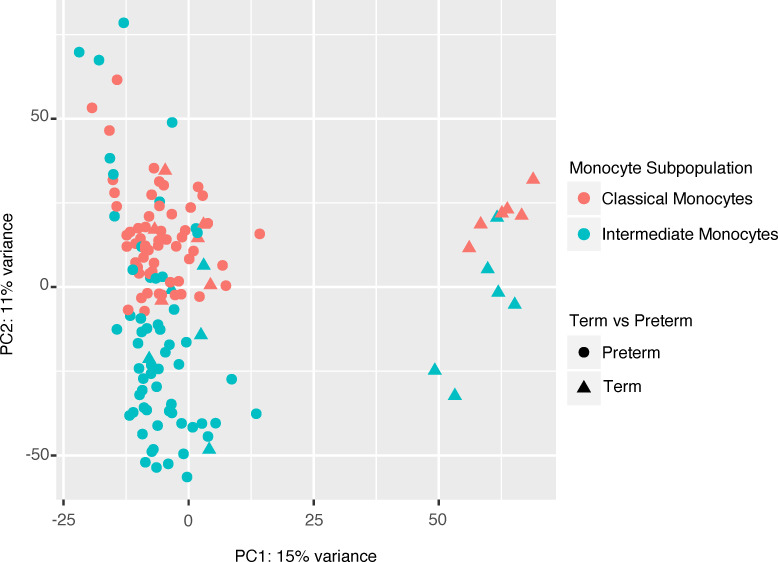
Principal component analysis of intermediate and classical cord blood monocytes global gene expression. A principal component analysis (PCA) of all samples (*n* = 130) illustrates GA (PC1, 15% variance) and cell type (PC2, 11% variance) and explains major variability within the data set.

We then asked whether differential activation of monocyte subsets was associated with different domains of placental pathology. We first isolated classical and intermediate monocyte subsets via FACS and performed transcriptomic profiling of 140 samples (70 classical and 70 intermediate monocyte samples) using bulk RNA-Seq. As expected, cell type (i.e., classical versus intermediate monocyte) and GA explained most of the variability within the data set (Figure 4).

### Gene expression signature of gestational age in monocytes in the classical subset.

**Figure 5:**
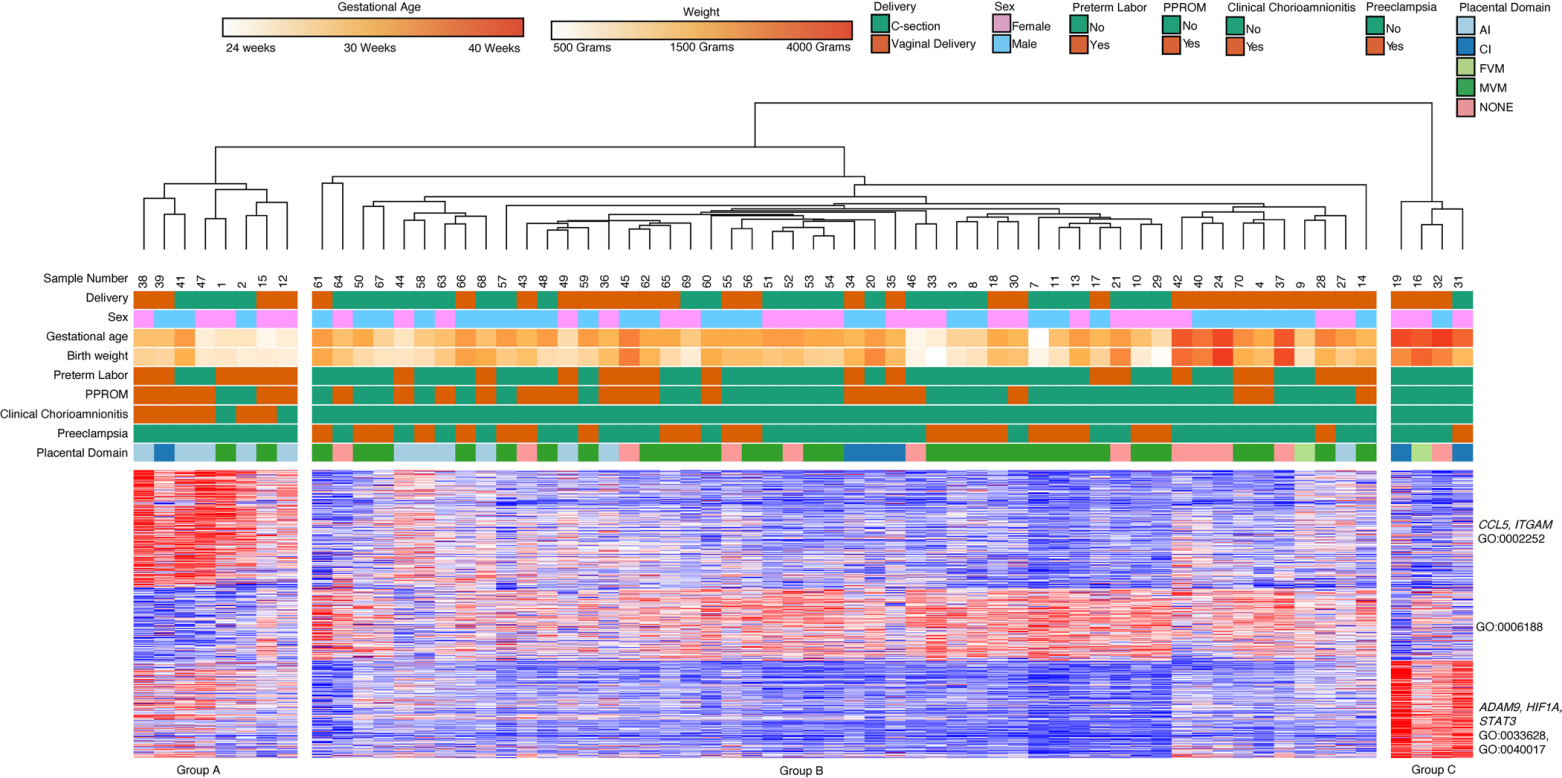
Classical monocyte gene expression clusters by GA, clinical phenotypes, and placental domains. Classical monocyte (*n* = 64) hierarchical clustering shows clustering primarily based on GA, placental inflammation, and placental vascular lesions. Cluster 1 included genes related to leukocyte activation, Cluster 2 contained genes involved in biosynthetic processes, and Cluster 3 contained genes related to cell adhesion and locomotion. There were 498 differentially expressed genes based on DeSeq2 using FDR < 0.05 and 2-fold change.

We then performed analysis on classical and intermediate monocyte subsets separately. Out of a total of 70 samples that were sequenced, 64 classical monocyte samples had sufficient quality for further analysis. Hierarchical clustering on subjects and k-means clustering on differential expressed genes identified 3 major clinical subgroups defined by differential gene expression across 3 clusters (Figure 5). We found that gestational age (GA), AI, and MVM lesions were the main factors separating clinical groups. Group A (8 subjects, median GA of 27 weeks, median BW of 1080 g) consisted primarily of samples collected from dyads with AI (75%) and chorioamnionitis (75%). Group B (52 subjects, median GA 33 weeks, median BW 1872.5 g) was characterized by MVM (*n* = 31, 62%) and preeclampsia (*n* = 22, 42%). Group C (4 subjects, median GA 39.5 weeks, median BW 2860 g) was characterized by term infants (100%).

Genes in Cluster 1 (200 genes) were upregulated in Group A, the group composed of samples with inflammatory exposure and included genes related to leukocyte activation based on Gene Ontology (GO) terms (*CCL5*, *ITGAM*; GO:000227, GO:0002252). Cluster 2 (128 genes) included genes involved in biosynthetic processes (GO:0006188). This cluster of genes was highly expressed in Group B but lower in Group A and C. Cluster 3 (170 genes) contained genes related to cell adhesion and locomotion (*ADAM9*, *HIF1A*, *STAT3*; GO:0033628, GO:0040017) and these genes were upregulated in Group C, which contained samples from term infants.

### There are differences in classical monocyte gene expression based on method and timing of delivery.

**Figure 6:**
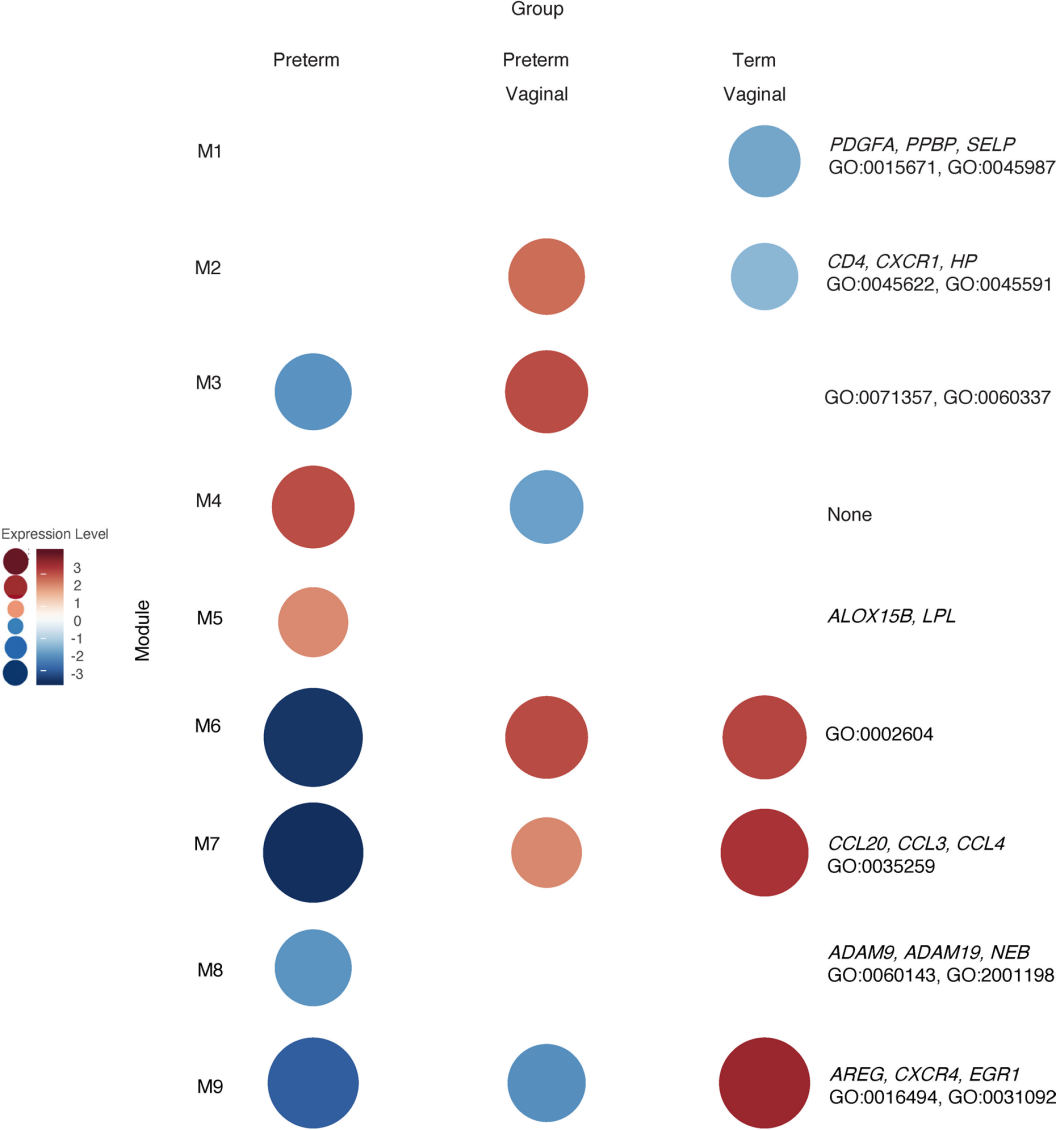
WGCNA of classical monocytes reveals gene clusters based on GA and mode of delivery. WGCNA of *n* = 64 samples revealed ontologies related to immune cell activation, migration, and chemokine response were unregulated in term vaginal deliveries (Module 9 [M9]). Genes related to inflammation and inflammatory response were upregulated in term vaginal and preterm vaginal deliveries and were relatively downregulated in preterm cesarean deliveries (M6 and M7). Genes related to neutrophil activity and granulocytes were upregulated in term preterm vaginal deliveries and downregulated in term vaginal deliveries (M2). Classical monocytes from preterm vaginal deliveries upregulated genes associated with DC interaction compared with monocytes isolated from preterm caesarean deliveries (M3).

The immune system plays a critical role in the normal maintenance of pregnancy and in the onset of parturition, while premature and abnormal parturition has been linked to immune cell dysfunction (19–23). Therefore, we analyzed the above samples (*n* = 64) based on timing (term versus preterm) and mode of delivery (cesarean section versus vaginal delivery). The preterm vaginal group (27 subjects, median GA 33, median BW 1990 g) had an older median GA and BW than the preterm cesarean group (30 subjects, median GA 31 weeks, median BW 1295 g). Both groups had subjects with preeclampsia (33% in the preterm vaginal group and 43% in the preterm cesarean group). Both groups had subjects with chorioamnionitis (56% in the preterm vaginal group and 10% in the preterm cesarean group). There were 6 term vaginal deliveries (6 subjects, median GA 39.5 weeks, median BW 3320 g) and 1 sample in the term cesarean section group (1 subject, 38 weeks, median BW 2090 g). The term cesarean section sample was from a mother in labor and with preeclampsia who proceeded to cesarean section due to fetal distress. While differential gene expression was unrevealing, we did find differences with weighted gene coexpression network analysis (WGCNA; Figure 6). Since the WGCNA method first identifies enriched modules and then assigns the module as being relatively increased or decreased within that sample group compared with the other, we performed the analysis with and without the single cesarean delivery sample included.

Specifically, the genes in Module 1 (115 genes) had lower expression levels in the term group and were linked to smooth muscle relaxation (*PDGFA*, *PPBP*, *SELP*; GO:0015671, GO:0045987). The genes in Module 2 (185 genes) were highly expressed in the preterm vaginal delivery group and had lower expression levels in the term vaginal delivery group. These genes were linked to interaction with T cells and T cell differentiation (*CD4*, *CXCR1*, *HP*; GO:0045622, GO:0045591). Module 2 did not have significantly different expression levels in the cesarean delivery group. Module 3 (23 genes) was upregulated in the preterm vaginal delivery group compared with the preterm cesarean delivery group, and it included genes involved in cellular response to type 1 IFN (GO:0071357, GO:0060337). Module 4 (24 genes) was upregulated in the preterm cesarean group compared with the preterm vaginal delivery group but without significant ontologies. Module 5 (35 genes) was the only module with higher levels of expression in the preterm cesarean section group compared with the term cesarean section group. The genes in this module included *ALOX15B* and *LPL*, but there were no enriched ontologies. Modules 6 (38 genes) and 7 (59 genes) had the lowest level of expression in the preterm cesarean group. The genes within this module were related to interactions with dendritic cells (DCs) (*CCL20*, *CCL3*, *CCL4*; GO:0002604). We found that Module 8 (60 genes) and Module 9 (687 genes) were highly expressed in the term group and relatively lower in the preterm group. The genes in this module were linked to chemokine receptors, platelet interactions, and DC activation (*ADAM9*, *ADAM19*, *AREG*, *CXCR4*, *EGR1*, *NEB*; GO:0060143, GO:2001198, GO:0016494, GO:0031092).

### Classical monocytes from preterm births upregulate genes related to leukocyte activation while downregulating genes related to protein translation.

**Figure 7:**
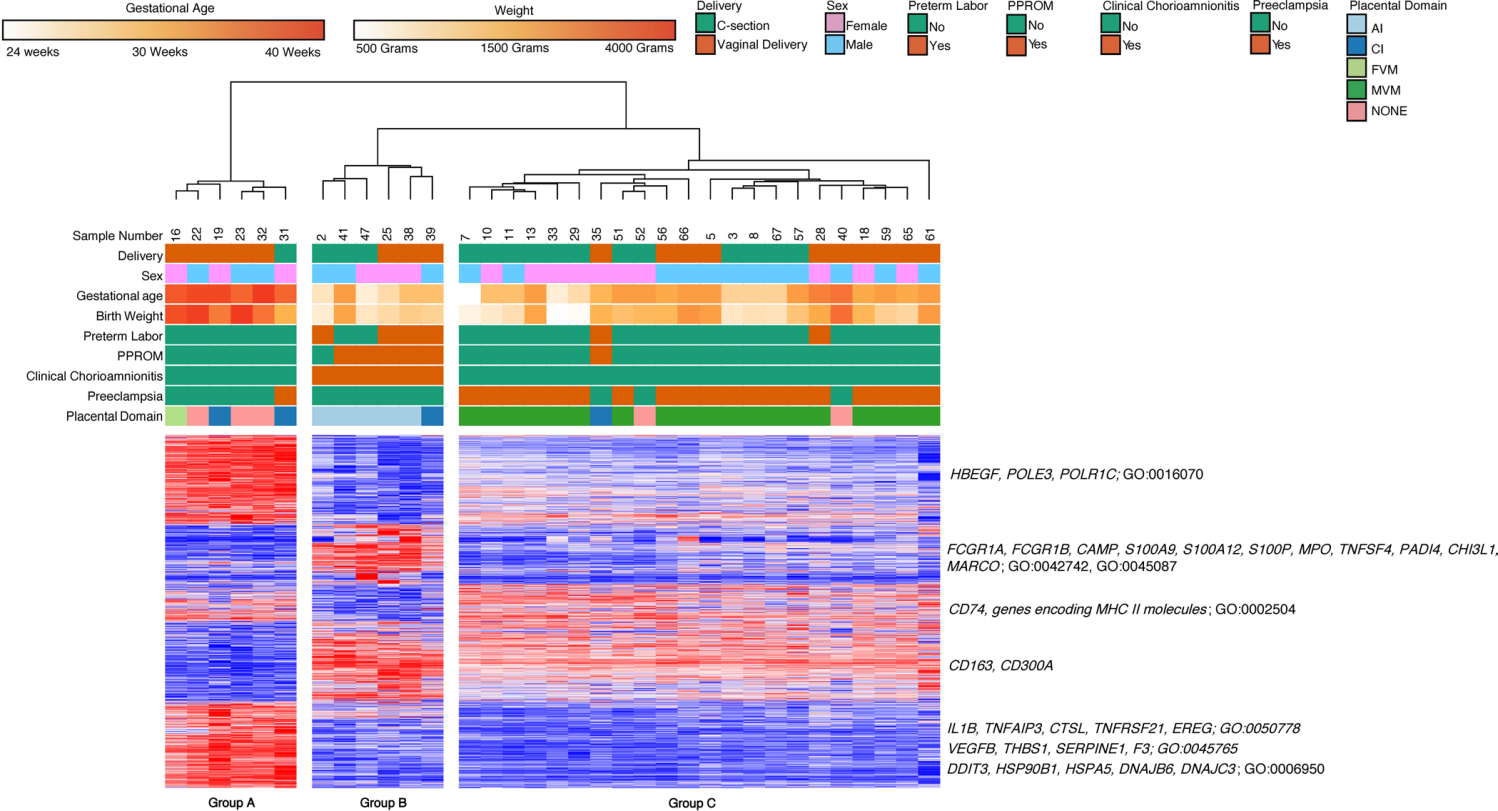
Placental domains and their associated clinical phenotypes lead to clustering of classical monocyte gene expression. Classical monocytes samples (*n* = 34) based on placental domain with the associated clinical phenotype, broken down into 3 hierarchical clusters. The groups comprised primarily of term infants (Group A), preterm infants with acute chorioamnionitis and placental inflammation (Group B), and preterm infants with placental vascular lesions and preeclampsia (Group C). There were 2219 differentially expressed genes based on an FDR < 0.05 and 2-fold change.

Since placental histopathology did separate our samples into different groups, we further refined our data by analyzing samples with AI, CI, and MVM with their associated clinical features (i.e., chorioamnionitis and preeclampsia) (24–27). We excluded preterm samples with FVM due to low sample numbers and lack of clear clinical association with this placental pathology. Specifically, we narrowed our analysis to samples with chorioamnionitis and AI on placental pathology, preterm labor or preterm delivery with CI, preeclampsia with MVM lesions, preterm infants with healthy placenta, and healthy term dyads. There were *n* = 34 samples that met these criteria. Hierarchical clustering on subjects and k-means clustering on highly variable genes in classical monocytes identified 3 clinical subgroups defined by differential gene expression across 5 clusters (Figure 7). Group A (6 subjects, median GA 39.5 weeks, median BW 3145 g) consisted of children born at term (100%). One subject in this group was born to a mother with preeclampsia, while others had no underlying prenatal conditions. Group B (6 subjects, median GA 29.5 weeks, median BW 1390 g) was characterized by prematurity, AI (83%), and chorioamnionitis (100%). Group C (22 subjects, median GA 33 weeks, median BW 1765 g) was characterized by prematurity, MVM lesions on placenta (86%), and preeclampsia (81%).

Genes in Cluster 1 (558 genes) were highly expressed in Group A but had lower expression in Group C and were downregulated in Group B. Based on GO terms, these genes were related to mRNA biosynthesis and metabolism (*HBEGF*, *POLE3*, *POLR1C*; GO:0016070). Genes in Cluster 2 (376 genes) were specifically upregulated in Group B and included genes related to cell migration (*SELL*, *ITGAM)*, inflammatory response and response to infection (*FCGR1A*, *FCGR1B*, *CAMP*, *S100A9*, *S100A12*, *S100P*, *MPO*, *TNFSF4*, *PADI4*, *CHI3L1*, *MARCO*; GO:0042742, GO:0045087), cell cycle, and negative regulation of myeloid cell differentiation (*MKI67*, *CDC14B*, *HIST1H3D*, *HIST1H3J*, *LILRB1*; GO:0007049; GO:0045638). Genes in Cluster 3 (234 genes) were upregulated in both Group A and Group C and were related to antigen processing and presentation (*CD74*, genes encoding MHCII molecules; GO:0002504). Genes in Cluster 4 (504 genes) were upregulated in both Groups B and C, the 2 groups composed primarily of samples obtained from preterm deliveries. While there was no significant GO biological process associated with this cluster of genes, it included several genes associated with monocyte activation and immune response (*CD163*, *CD300A*, and others). Genes in Cluster 5 (547 genes) were specifically expressed in Group A and included genes related to immune response (*IL1B*, *TNFAIP3*, *CTSL*, *TNFRSF21*, *EREG*; GO:0050778), regulation of angiogenesis (*VEGFB*, *THBS1*, *SERPINE1*, F3; GO:0045765), molecular chaperones and adaptation to stress (*DDIT3*, *HSP90B1*, *HSPA5*, *DNAJB6*, *DNAJC3*; GO:0006950), and monocyte differentiation and maturation (*CEBPB*, *BHLHE40*).

### WGCNA shows enrichment of modules unique to term and preterm groups in classical monocytes.

**Figure 8:**
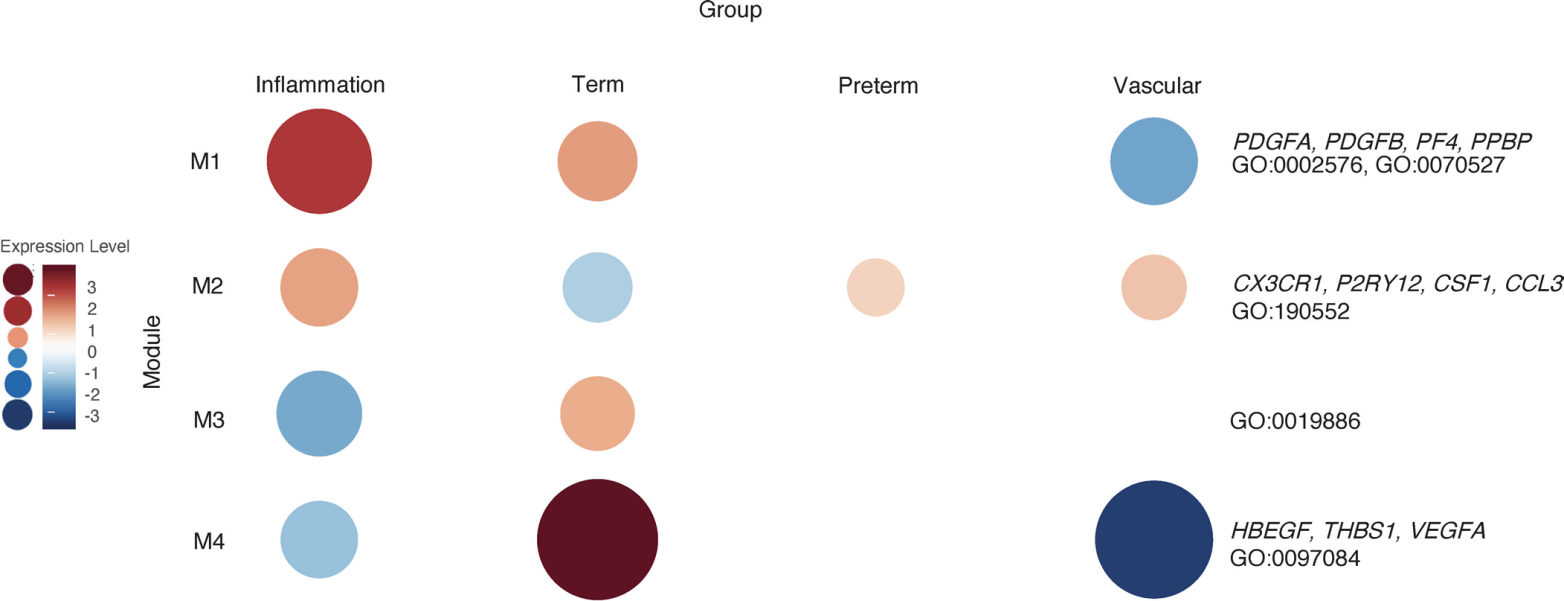
WGCNA of placental domains and their associated clinical phenotypes reveal unique cluster of classical monocyte gene expression. WGCNA of classical monocyte samples (*n* = 34) with available placental pathology and pregnancies complicated by clinical findings associated with that placental pathology reveals modules that are unique to each group. Genes related to platelet interactions were present in Module 1 (M1) and highly expressed in the inflammation and term groups. M2 contained genes related to inflammatory cytokines based on GO biological processes that were relatively upregulated in the preterm group. Genes related to antigen presentation were found in M3 and were relatively upregulated in the term group. Genes related to processes like artery development, such as *VEGFA*, were expressed in term neonates but were lower in preterm neonates exposed to placental pathology.

We then used the same samples as above (*n* = 34) and performed a WGCNA to validate our hierarchical clustering data and look for relevant genes that would be missed by other methods. WGCNA for classical monocytes revealed 4 gene network modules (Figure 8). Module 1 (136 genes) contained genes highly expressed in samples from term infants and preterm infants exposed to inflammation compared with samples from preterm infants. These genes were closely associated with platelet degranulation and platelet aggregation (*PDGFA*, *PDGFB*, *PF4*, *PPBP*, *SELP*; GO:0002576, GO:0070527). Module 2 (788 genes) was highly expressed in all preterm groups relative to term groups. The genes in the module were associated with GO biological processes related to monocyte activation and migration (*CX3CR1*, *P2RY12*, *CSF1*, *CCL3*; GO:190552). The genes in Module 3 (48 genes) were related to antigen processing (GO:0019886) and were highly expressed in samples obtained from term infants compared with preterm infants. Module 4 (87 genes) contained genes related to vascular smooth muscle development (*THBS1*, *VEGFA*; GO:0097084) and were highly expressed in samples from term infants compared with preterm infants with known placental pathology.

### Two major gene expression clusters differentiate AI and MVM in classical monocytes.

**Figure 9:**
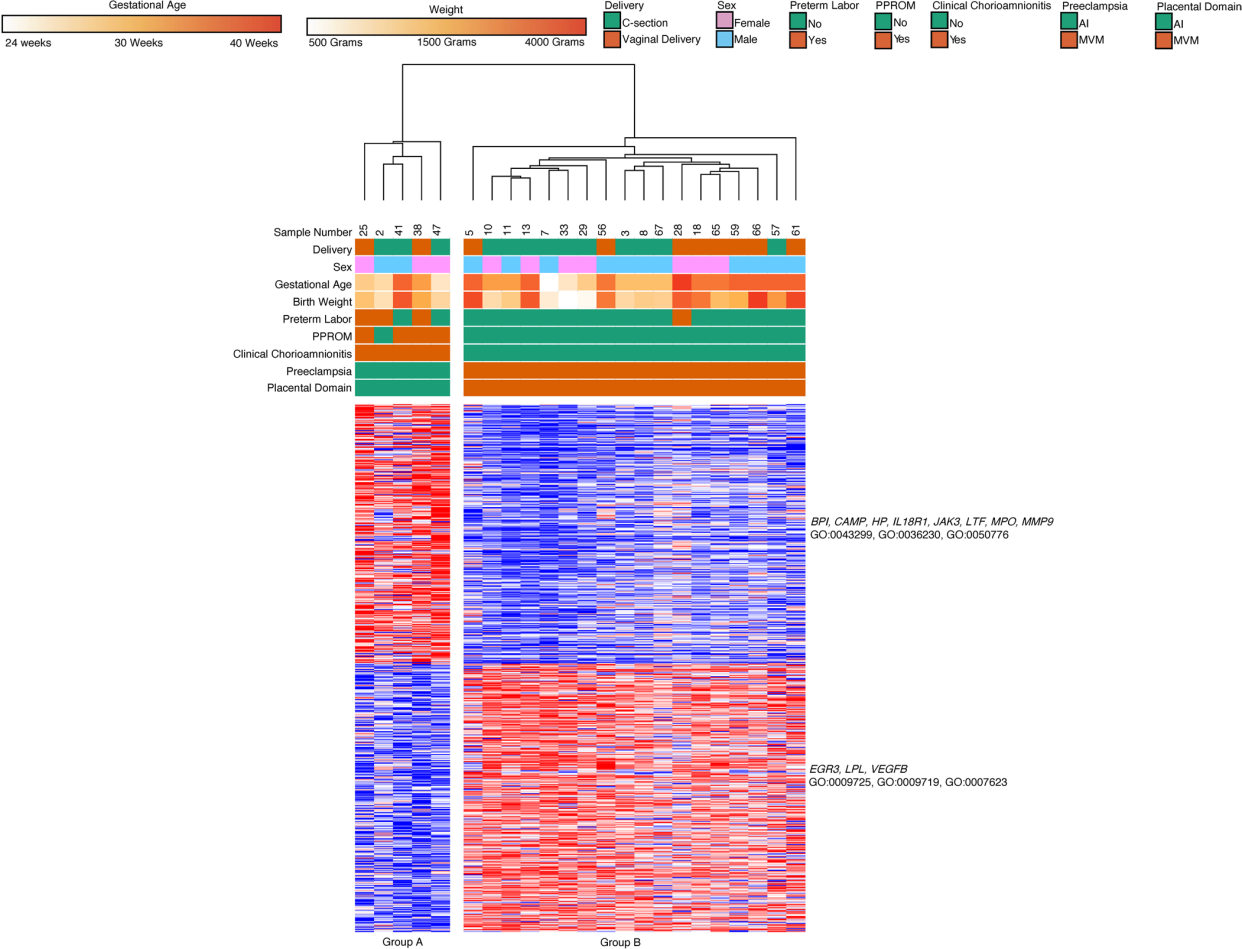
Gene expression of classical monocytes from preterm dyads cluster based on associated clinical findings and placental pathology. Classical monocytes (*n* = 23) with known placental pathology and associated clinical features were divided into 2 hierarchical clusters. Group A was composed of preterm deliveries with chorioamnionitis and placental inflammation. Group B was composed of preterm deliveries complicated by preeclampsia MVM lesions. Genes related to granulocyte function were upregulated in Group A, while genes related to cell proliferation were upregulated in Group B. Based on an FDR < 0.05 and 2-fold change, there were 598 differentially expressed genes using DeSeq2.

As placental pathologies can coincide, we refined our dataset to include only subjects with placental pathologies and their associated clinical phenotype (28). For this analysis, samples with unknown placental pathologies were excluded. Additionally, we excluded term infants, as many lacked placental pathologies or had low degree of placental pathology without clinical correlation. We also excluded samples where MVM and AI were both present. Analysis of these samples (*n* = 23) led to 2 major groups based on hierarchical clustering (Figure 9). Group A primarily had subjects with AI (*n* = 5, median GA 29 weeks, median BW 1310 g), and Group B had infants with MVM (*n* = 18, median GA 33 weeks, median BW 1440 g).

We found 2 major gene clusters based on k-means clustering of genes in the analysis. Cluster 1 (294 genes) was upregulated in Group A, while Cluster 2 (304 genes) was upregulated in Group B. Cluster 1 contained genes related to leukocyte activation and degranulation (*BPI*, *CAMP*, *HP*, *IL18R1*, *JAK3*, *LTF*, *MPO*, *MMP9*: GO:0043299, GO:0036230, GO:0050776). Cluster 2 contained genes related to positive regulation of cell proliferation and response to hormone stimulus (*EGR3*, *LPL*, *VEGFB*; GO:0009725, GO:0009719, GO:0007623). There were no significant differences in gene expression or WGCNA based on mode of delivery in this sample set.

### Clustering of intermediate monocyte gene expression data reveals unique k-means clusters based on placental domains and clinical features.

**Figure 10:**
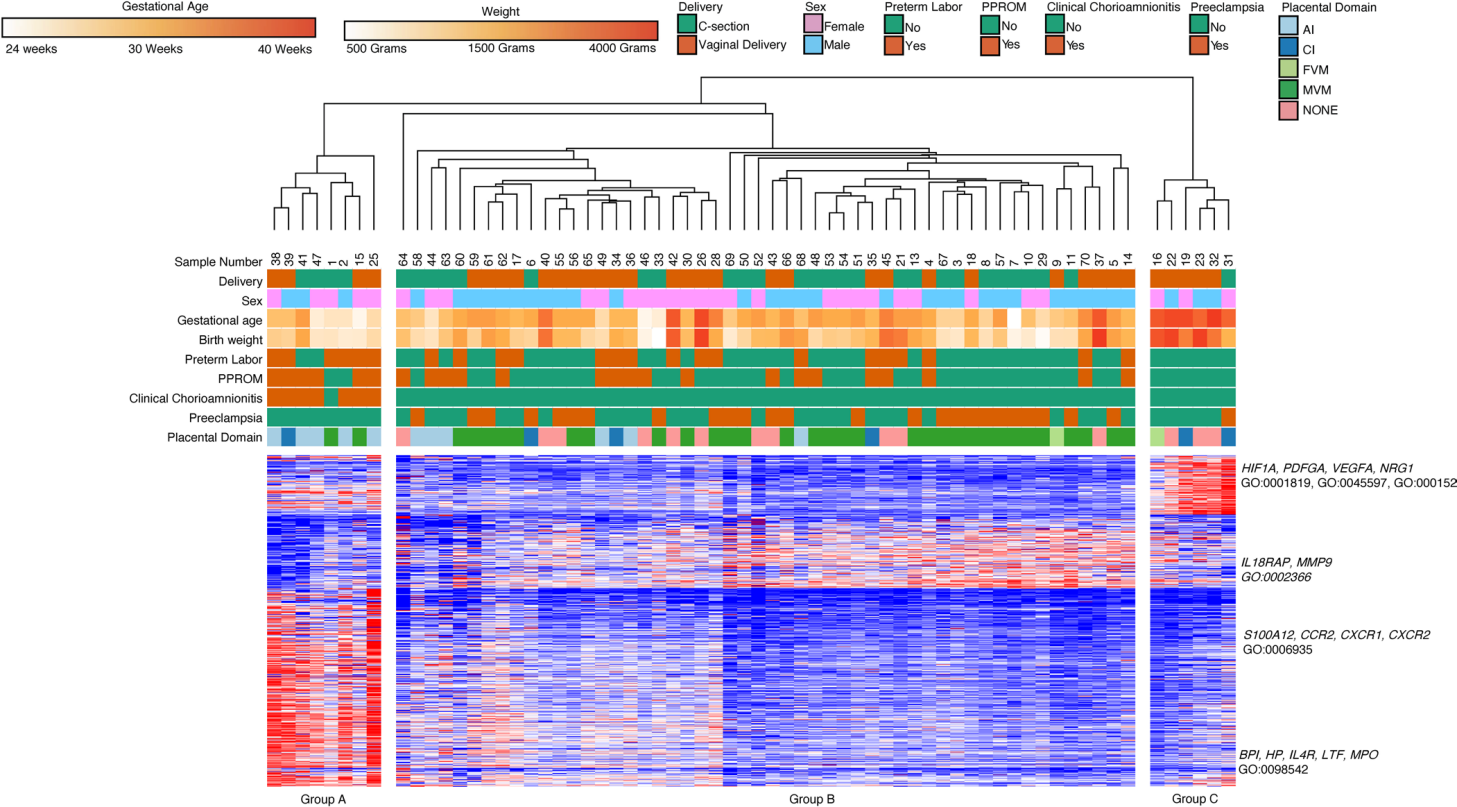
Intermediate monocyte gene expression clusters by GA, clinical phenotypes, and placental domains. Similar to classical monocytes, intermediate monocyte samples (*n* = 66) show hierarchical clustering based primarily based on GA. Cluster 1 included genes related to angiogenesis and cytokine production. Cluster 2 contained genes involved in biosynthetic processes, and Cluster 3 contained genes related to leukocyte activation, chemotaxis, and defense response. Differentially expressed genes were assessed with DeSeq2 using FDR < 0.05 and 2-fold change.

We then performed a similar unbiased hierarchical clustering analysis of all intermediate monocyte gene expression data (*n* = 66) and k-means clustering of gene expression data (Figure 10). There were additional samples in this group compared with the classical monocyte samples because of RNA-Seq quality. We again found that GA, AI, and MVM were the main factors separating clinical groups. Group A (8 subjects, median GA 27 weeks, median BW 1080 g) consisted primarily of samples collected from dyads with AI (75%) and chorioamnionitis (87.5%). Group B (52 subjects, median GA 33 weeks, median BW 1872.5 g) was characterized by MVM (*n* = 31, 62%) and preeclampsia (*n* = 22, 42%). Group C (6 subjects, median GA 39.5 weeks, median BW 2145 g) was characterized by term infants (100%).

Our analysis revealed 3 major clusters, which were then analyzed for significant GO terms. Cluster 1 (89 genes) contained genes related to cell differentiation (GO:0045597), cytokine production (GO:0001819), and angiogenesis (GO:0001525) and were highly expressed in Group C, were less expressed in Group A, and had low levels of expression in Group B. The genes in this cluster included *HIF1A*, *PDFGA*, *VEGFA*, and *NRG1*. Cluster 2 (110 genes) did not contain statistically significant biological processes. This cluster had low levels of expression in Group A compared with B and C. Cluster 3 contained genes related to leukocyte activation (*BPI*, *IL18RAP*, *MMP9*; GO:0002366), chemotaxis (*S100A12*, *CCR2*, *CXCR1*, *CXCR2*; GO:0006935), and defense response (*BPI*, *HP*, *IL4R*, *LTF*, *MPO*; GO:0098542). This cluster was highly expressed in Group A, with lower levels of expression in Group B, and with the lowest levels of expression in Group C.

### Gene expression modules within intermediate monocytes are influenced by mode and timing of delivery.

**Figure 11:**
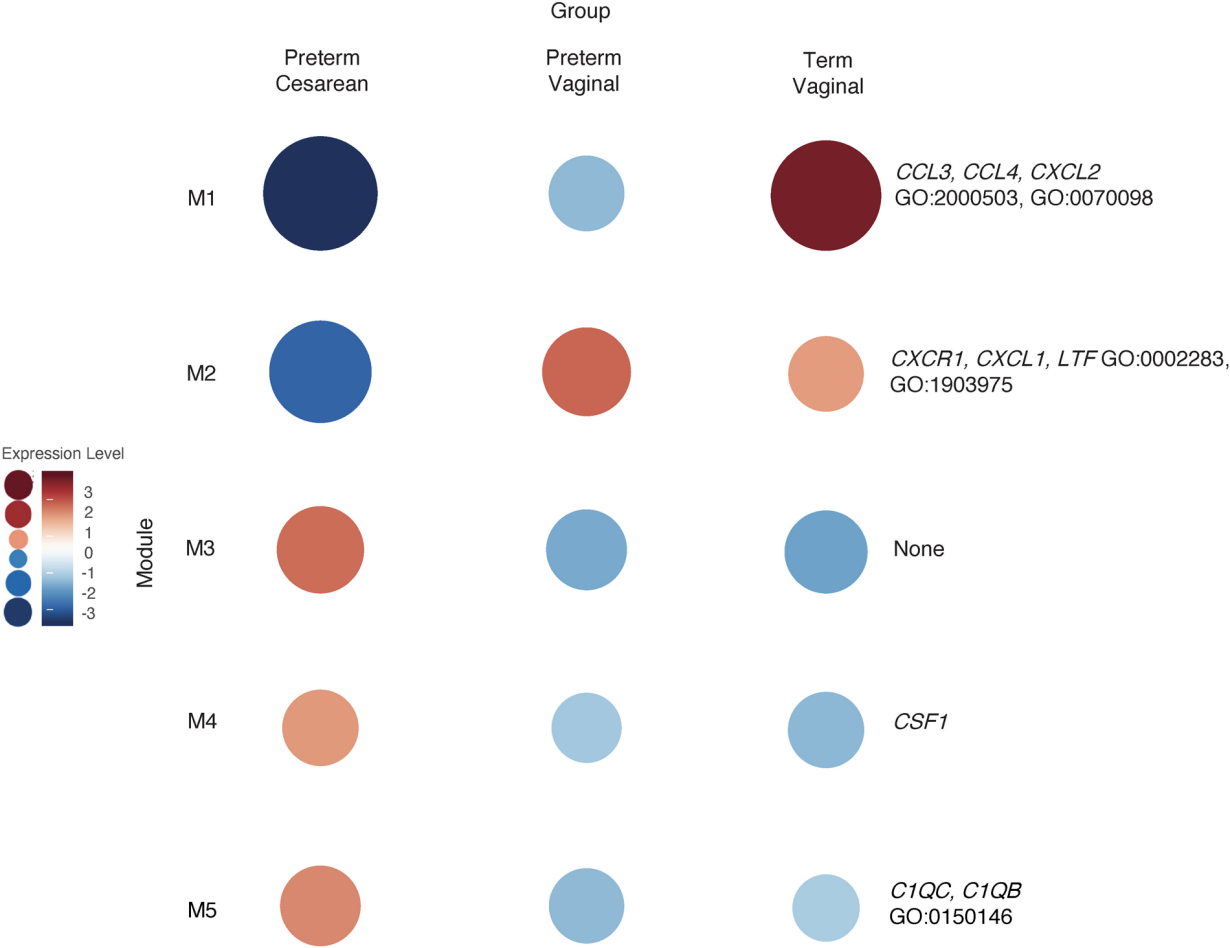
WGCNA of intermediate monocytes reveals gene clusters based on GA and mode of delivery. WGCNA analysis of intermediate monocyte gene expression, like classical monocytes, showed that genes related to chemokine signaling were upregulated in term deliveries (M1). Genes related to immune cell migration were highly expressed the vaginal delivery group (M2). The gene ontologies in this module were like those in classical monocytes obtained from vaginal deliveries (Figure 6; M7) and were most highly expressed in the preterm vaginal delivery group.

Like the classical monocyte subset, we analyzed the above intermediate monocyte samples (*n* = 66) based on timing (term versus preterm) and mode of delivery (cesarean section versus vaginal delivery). As with the classical monocytes, the preterm vaginal group (27 subjects, median GA 33 weeks, median BW 1990 g) had an older median GA and BW than the preterm cesarean group (30 subjects, median GA 31 weeks, median BW 1295 g). Both groups had subjects with preeclampsia and chorioamnionitis (33% and 56%, respectively, in the preterm vaginal delivery group; 43% and 10%, respectively, in the preterm cesarean group). There were 8 term vaginal deliveries (8 subjects, median GA 39.5 weeks, median BW 3482.5 g) and 1 sample in the term cesarean section group (1 subject, 38 weeks, median BW 2090g, growth-restricted with preeclampsia). This sample was from a mother in labor who proceeded to cesarean section due to fetal distress. We again used WGCNA to highlight gene expression patterns unique to each group (Figure 11).

Module 1 was upregulated in the term delivery group compared with the preterm delivery group. The genes (65 genes) in this module were related to immune cell chemotaxis and chemokine-mediated signaling (*CCL3*, *CCL4*, *CXCL2*, *IL1B*, and *VEGFA*; GO:2000503, GO:0070098). Module 2 was downregulated in the preterm cesarean delivery group compared with the other 2 groups and contained genes (290 genes) related to cell migration based on ontologies (*CXCR1*, *CXCL1*, *LTF*; GO:0002283, GO:1903975). Modules 3 (130 genes) and 4 (194 genes) did not contain significant GO enrichment in these groups. Module 5 (588 genes) contained genes and ontologies related to complement function and interaction with cell junctions (*C1QC*, *C1QB*; GO:0150146). This module had increased expression in the preterm cesarean delivery group compared with the vaginal delivery groups.

### Placental histology and clinical data reveal gene expression patterns unique to perinatal exposure prior to delivery.

**Figure 12:**
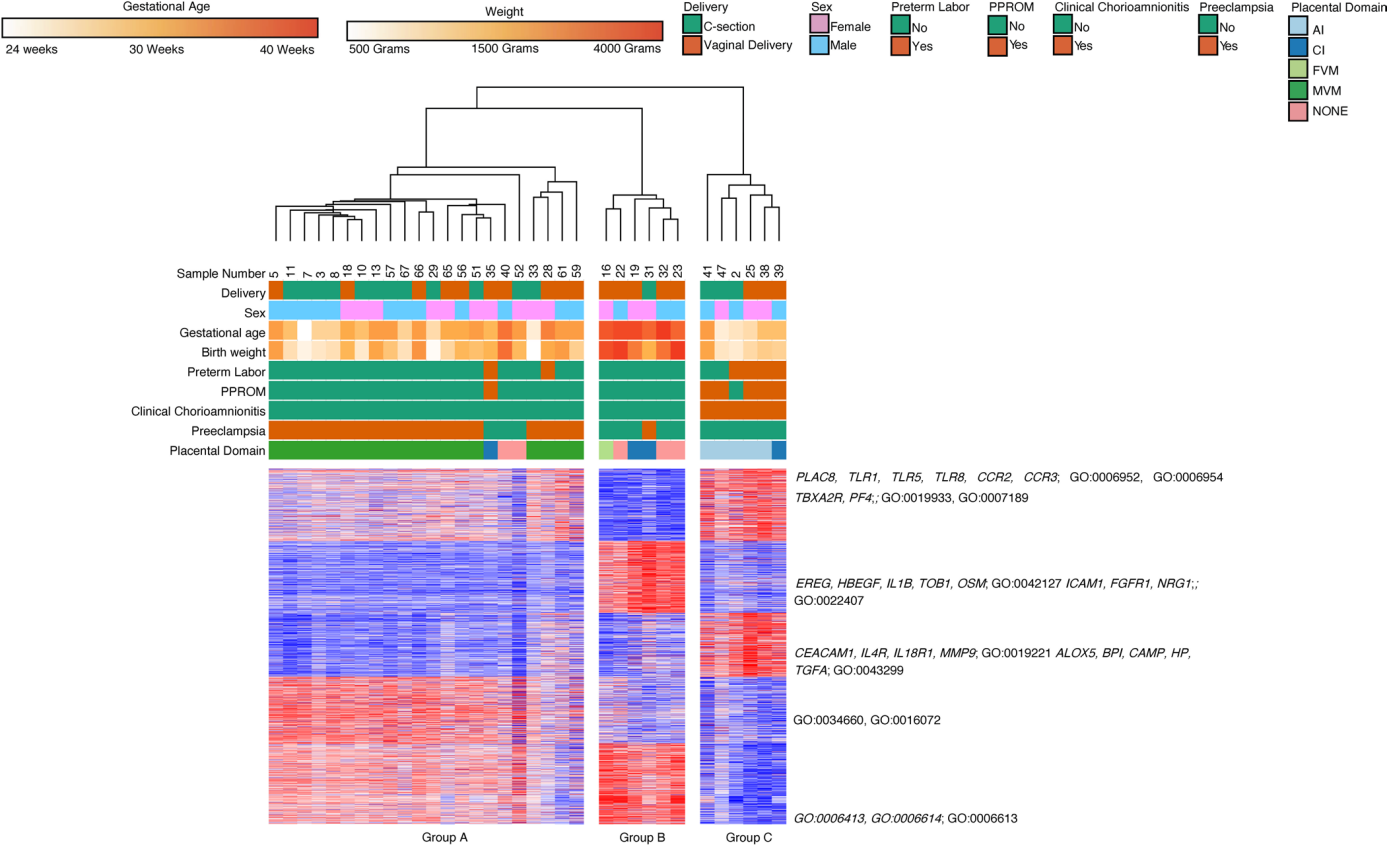
Placental domains and their associated clinical phenotypes lead to clustering of intermediate monocyte gene expression. There were 3 major groups based on hierarchical clustering of intermediate monocyte gene expression (n = 66). The groups were composed primarily of term infants (Group B), preterm infants with acute chorioamnionitis and placental inflammation (Group C), and preterm infants with MVM and preeclampsia (Group A). There were 3549 differentially expressed genes based on DeSeq2, using FDR < 0.05 and 2-fold change. The genes in Cluster 1 were involved in inflammatory biological processes and cell signaling. The genes in Cluster 2 were involved in cell proliferation and cell adhesion. Cluster 3 contained genes related to cytokine signaling and leukocyte degranulation. Cluster 4 and Cluster 5 contained genes involved in transcription and translational processes.

We again refined our data set by taking comparing placental pathologies and their associated clinical features. The same *n* = 34 samples as the classical monocytes met these criteria. Hierarchical clustering on subjects and k-means clustering on differentially expressed genes identified 3 clinical subgroups defined by differential gene expression across 5 k-means clusters (Figure 12). Group A (22 subjects, median GA 33 weeks, median BW 1765 g) was characterized by prematurity, MVM lesions on placenta (86%), and preeclampsia (81%). Group B (6 subjects, median GA 39.5 weeks, median BW 3145 g) consisted of children born at term (100%). Only 1 subject in this group was born from a mother with preeclampsia, while others had no major comorbidity. Group C (6 subjects, median GA 29.5 weeks, median BW 1390 g) was characterized by prematurity, AI (83%), and chorioamnionitis (100%).

Cluster 1 (706 genes) was highly expressed in Group C, the inflammatory group, with lower levels of expression in Group A, and with the lowest levels of expression in Group B. Based on GO terms, the genes in this group were associated with biological processes involved in inflammatory response (*PLAC8*, *TLR1*, *TLR5*, *TLR8*, *CCR2*, *CCR3;* GO:0006952 and GO:0006954) and cell signaling (*TBXA2R*, *PF4*; GO:0019933 and GO:0007189). Cluster 2 (722 genes) was highly expressed in Group B, the term group, with lower levels of expression in both preterm groups. The genes in this cluster were associated with cell proliferation (*EREG*, *HBEGF*, *IL1B*, *TOB1*, *OSM*; GO:0042127) and cell adhesion (*ICAM1*, *FGFR1*; GO:0022407). Cluster 3 (644 genes) was highly expressed in Group C with lower levels of expression in Group A and B. The majority of the genes in this cluster were involved in cytokine-mediated signaling (*CEACAM1*, *IL4R*, *IL18R1*, *MMP9*, *TGFA*; GO:0019221), leukocyte degranulation (*ALOX5*, *BPI*, *CAMP*, *HP*; GO:0043299), and cell signaling (*NRG1*, *TGFA*; GO:0006935). Cluster 4 (668 genes) was highly expressed in the MVM group (Group A), with lower levels of expression in the healthy term group (Group B), and with the lowest levels of expression in the AI (Group C). Based on GO biological processes, the genes in this cluster were primarily related to ribosomal biogenesis and RNA processing (GO:0034660, GO:0016072). Cluster 5 (809 genes) was highly expressed in the preterm group (Groups A and B) with lower levels of expression in the term group (Group C). The cluster contained genes primarily involved in protein translation and trafficking (GO:0006413, GO:0006614, GO:0006613).

### WGCNA reveals enrichment of modules unique to term and preterm groups in intermediate monocytes.

**Figure 13:**
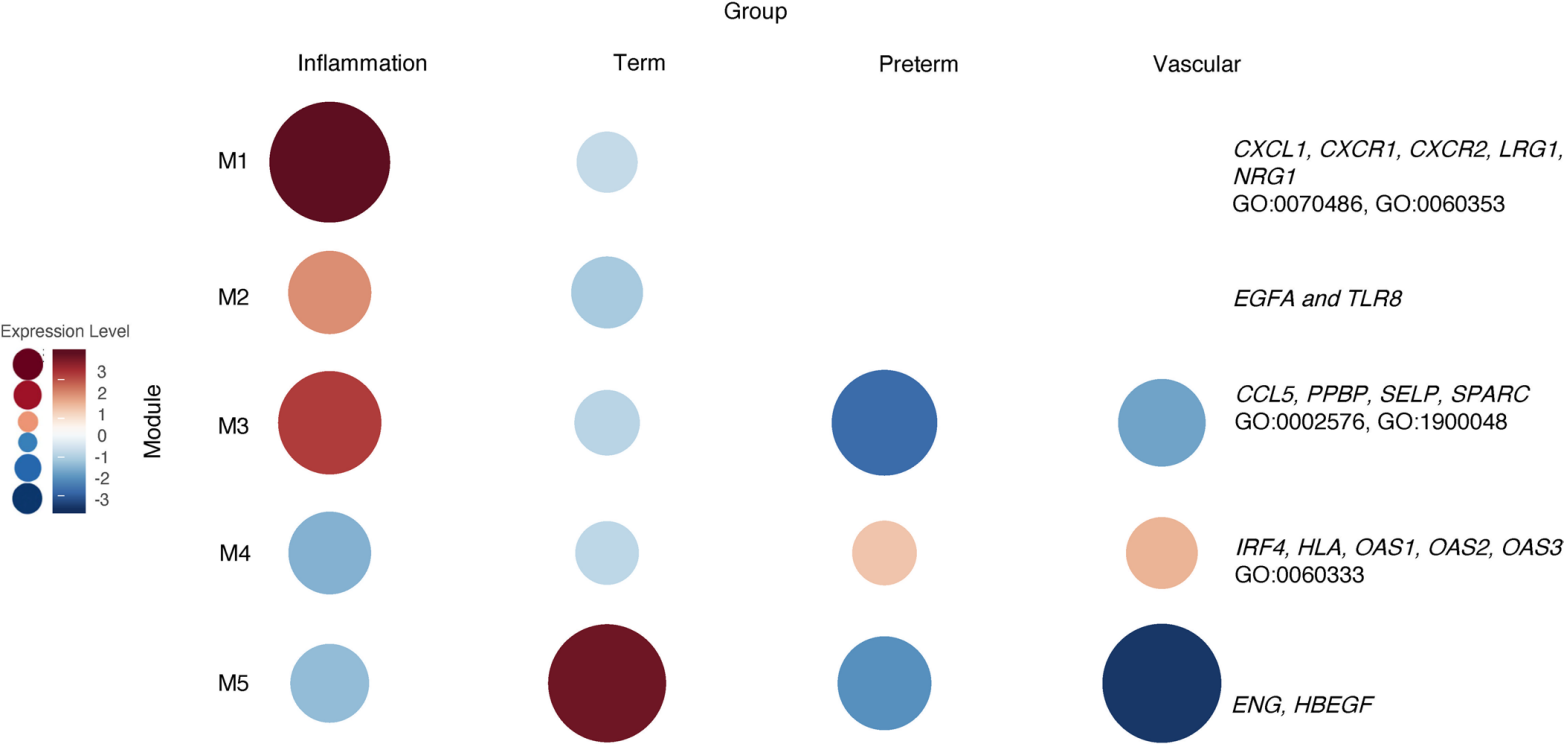
WGCNA of placental domains and their associated clinical phenotypes reveal unique cluster of intermediate monocyte gene expression. WGCNA of intermediate monocytes samples (*n* = 34) with available placental pathology and pregnancies complicated by clinical findings associated with that placental pathology reveals 5 modules. The genes Module 1 (M1), M2, and M3 were highly expressed in the inflammation group. The genes in this group were associated with biological processes involved in monocyte chemotaxis, cell adhesion, and hemostasis. M4 had higher levels of expression in intermediate monocytes from the preterm group with vascular lesions and healthy premature infants. These genes were related to IFN signaling and signal transduction.

We then used the same samples (*n* = 34) to perform a WGCNA on the intermediate monocyte gene expression data, and our analysis revealed 5 gene expression modules (Figure 13). Module 1 (150 genes) and 2 (221 genes) were significantly increased in the inflammatory group and decreased in the term group. Module 1 contained genes related to leukocyte degranulation, monocyte chemotaxis, cell adhesion, and inflammatory response (*CXCL1*, *CXCR1*, *CXCR2*, *LRG1*, *NRG1*, *S100A9*; GO:0070486, GO:0060353, GO:0090026). Module 2 did not contain significant ontology terms but included genes such as *VEGFA* and *TLR8*. Module 3 (38 genes) was unique to intermediate monocytes exposed to inflammation when compared with the other groups and contained GO terms related to platelet function, hemostasis, and wound healing (*CCL5*, *PPBP*, *SELP*, *SPARC*; GO:0002576, GO:1900048, GO:0061041). Module 4 (642 genes) was the only module significantly increased in the MVM group and relatively decreased in the inflammatory, term, and preterm group. This module contained genes related to interferon signaling (*IRF4*, *HLA*, *OAS1*, *OAS2*, *OAS3*; GO:0060333). Module 5 (56 genes) contained genes significantly increased in the term group but decreased in the 3 preterm groups. There were no significant biological processes associated with the genes in this module.

### Two major gene expression clusters differentiate AI and MVM in intermediate monocytes.

**Figure 14:**
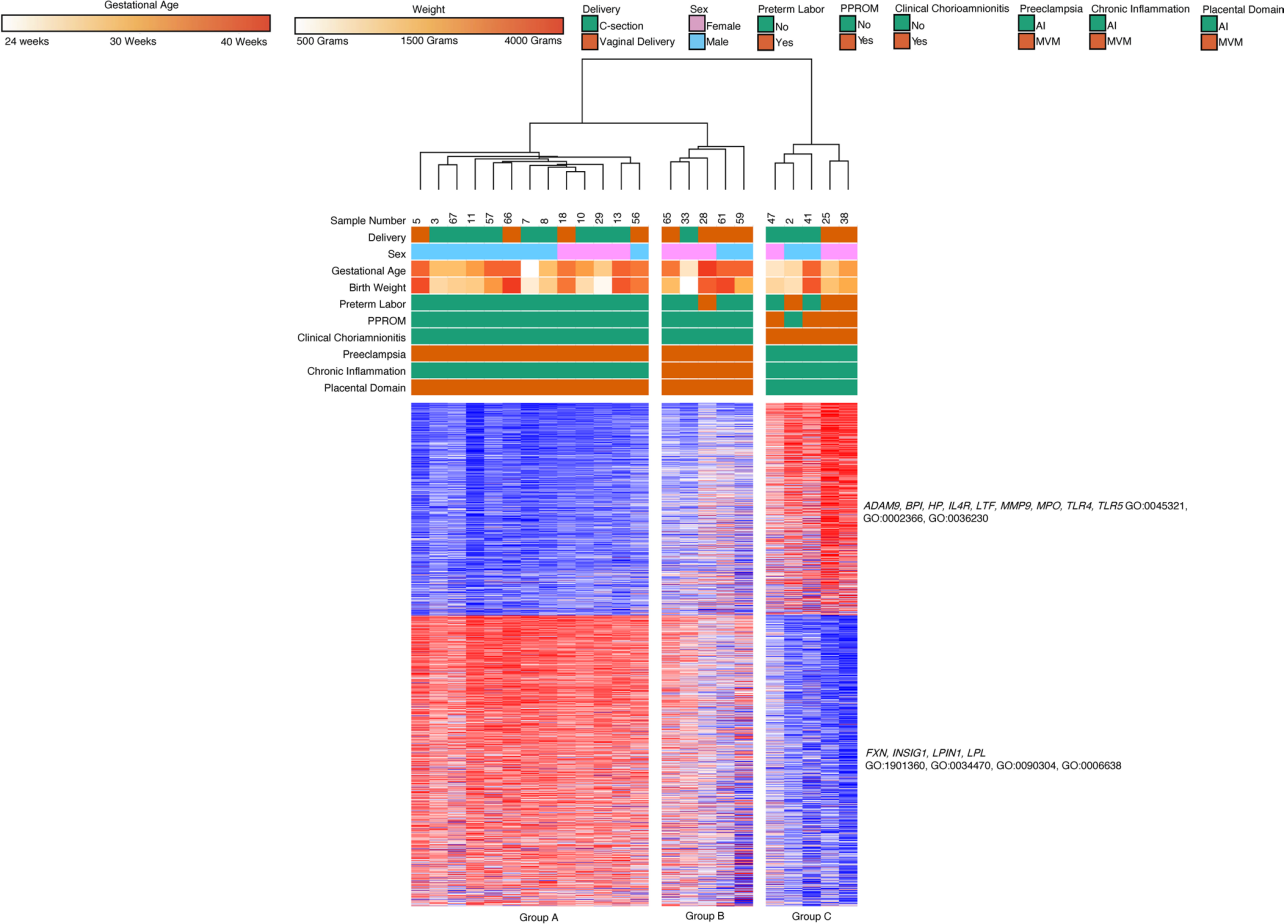
Chronic placental inflammation with preeclampsia leads to unique clustering of intermediate monocyte gene expression. Intermediate monocytes (*n* = 23) with known placental pathology and associated clinical features also divided into 3 hierarchical clusters. Group A was composed of preterm deliveries complicated by preeclampsia and placental vascular lesions. Group B was composed of preterm deliveries complicated by preeclampsia and placental vascular lesions, and this group’s patients also had high degree chronic inflammatory lesions. Group C was composed of preterm deliveries with chorioamnionitis and placental inflammation. Genes related to leukocyte activity, function, migration, and degranulation were upregulated in Group C. Genes related to lipid metabolism and organic cyclic compound processing were upregulated in Groups A and B. There were 1852 differentially expressed genes based on an FDR < 0.05 and 2-fold change using DeSeq2.

We performed a similar analysis to the classical monocyte subset by excluding samples with unknown placental pathologies, term infants, and cooccurring MVM and AI. Gene expression analysis of the samples (*n* = 23) that met the above criteria led to 3 major groups based on hierarchical clustering (Figure 14). Group A had infants with MVM and preeclampsia (*n* = 13, median GA 31 weeks, median BW 1200 g), while Group C had subjects with AI and chorioamnionitis (*n* = 5, median GA 28 weeks, median BW 1310 g). Though our data initially broke down into 2 hierarchical clusters, based on k-means clustering of genes, we found that the MVM group broke down into 2 subgroups. Group B contained subjects with preeclampsia and placental pathology with MVM and high degree CI (*n* = 5, median GA 34 weeks, median BW 1500 g).

There were 2 major gene clusters based on k-means clustering. Cluster A was upregulated in Group C and included genes (787 genes) related to leukocyte activity and activation (*ADAM9*, *BPI*, *HP*, *IL4R*, *LTF*, *MMP9*, *MPO*, *TLR4*, *TLR5*; GO:0045321, GO:0002366, GO:0036230). Cluster 2 was upregulated in Group A and Group B. The cluster contained genes (1065 genes) related to noncoding RNA processing, lipid metabolism, and organic cyclic compound processing (*FXN*, *INSIG1*, *LPIN1*, *LPL*; GO:1901360, GO:0034470, GO:0090304, GO:0006638). As with the classical monocyte samples, we found no significant differences in gene expression or WGCNA based on mode of delivery. There were no major clusters differentiating Group A from Group B based on k-means clustering iterations.

### Nonclassical monocytes potentially express genes related to leukocyte interaction with vascular endothelium.

The nonclassical monocyte population (*n* = 32) was difficult to sequence. However, we did obtain sufficient sequence quantity and quality output from 8 samples. The successful samples had significantly more cells (8 samples; mean 10,525 cells; SD 6668.4 cells), and this led to higher RNA yield (4749.4 picograms, SD 5168.5 picograms) compared with sample that failed (*n* = 24), which had fewer cells (2407.1 cells, SD 2626.8 cells) and lower RNA yield (1035.9 picograms, SD 1106.7 picograms).

**Figure 15:**
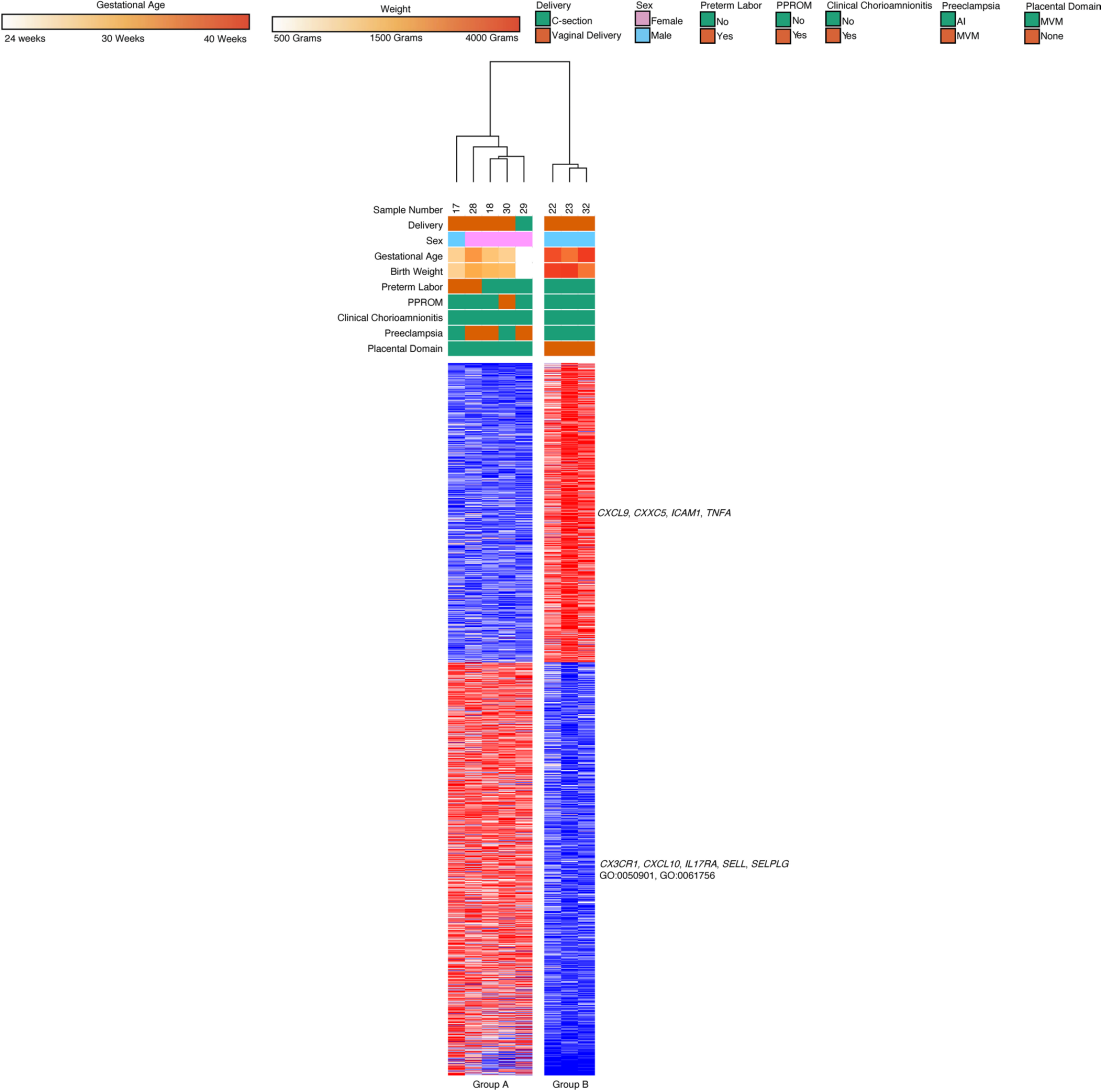
Nonclassical monocyte gene expression clusters by GA. Nonclassical monocytes (*n* = 24) divided into 2 major hierarchical cluster. One cluster was composed primarily of samples obtained from preterm deliveries with MVM on placental pathology (Group A). The second cluster contained samples from term deliveries with presumed normal placental pathology (Group B). Based on DeSeq2, 1938 genes were differentially expressed using FDR < 0.05 and 2-fold change. There were 2 major gene clusters based on k-means clustering. The genes in Cluster B were upregulated in Group A, and these genes were related to leukocyte adhesion to vascular endothelial cells and leukocyte tethering.

Differential gene analysis led to 2 significant clusters based on GA (Figure 15). Group A contained preterm infants with MVM lesions on placental pathology (5 subjects, median GA 32 weeks, median BW 1645g, 4 female infants, 4 vaginal deliveries, 3 mothers with preeclampsia), while Group B contained male term infants without any placental lesions (3 subjects, median GA 39 weeks, median BW 3378g, 3 male infants, 3 vaginal deliveries).

There were 2 major gene clusters. Cluster A (812 genes) was downregulated in Group A, and the genes in this group included *CXCL9*, *CXXC5*, *ICAM1*, and *TNFA*. There were no significant ontologies linked to the genes in this cluster. The genes in Cluster B (1126 genes) were upregulated in Group A. The genes in this group included *CX3CR1*, *CXCL10*, *IL17RA*, *SELL*, *SELPLG*, and various *TLR*s. These genes were related to leukocyte adhesion to vascular endothelial cells and leukocyte tethering (GO:0050901, GO:0061756).

### Multiplex immunoassay of plasma analytes reveals that classical and intermediate monocytes may be a source of plasma cytokines, chemokines, and growth factors.

From the above monocyte subjects, *n* = 24 had archived plasma for multiplex measurement of 30 analytes. There were 7 samples that correlated with inflammatory lesions on placenta (median GA 32 weeks, median BW 2040 g). Of these 7 samples, 57% (*n* = 4) had clinical chorioamnionitis, while 14% (*n* = 1) had preeclampsia. Four samples were from infants without any placental lesions (median GA 39 weeks, median BW 3247 g). These infants had no clinical chorioamnionitis or preeclampsia. The remaining samples (*n* = 13) correlated with placental MVM lesions. Of these, none had clinical chorioamnionitis, while 85% (*n* = 11) had preeclampsia.

**Figure 16:**
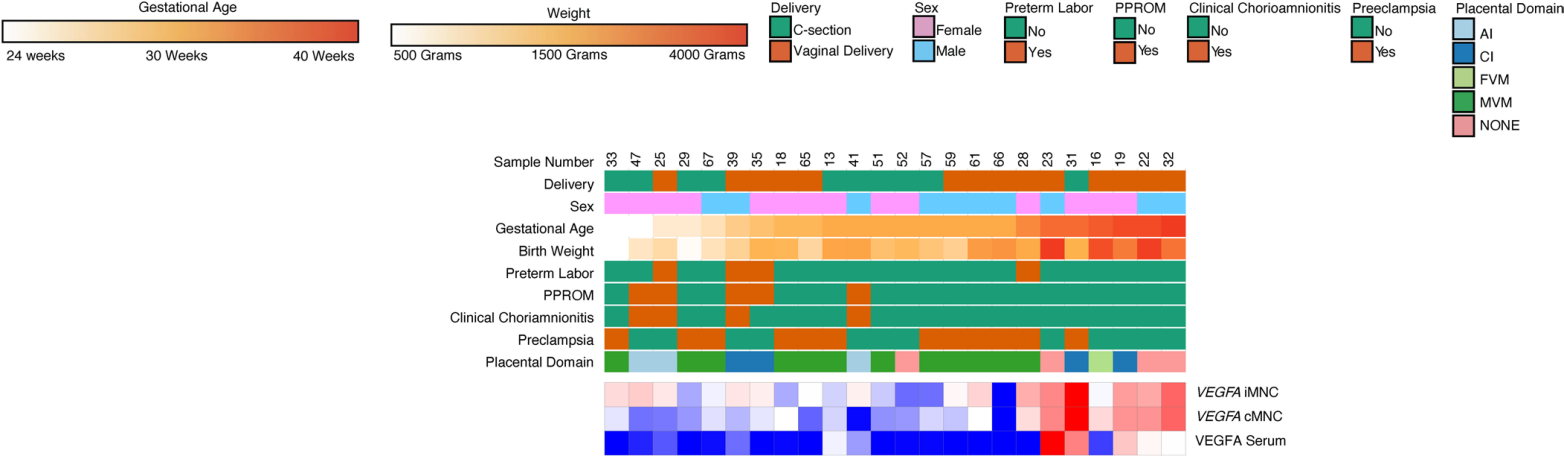
VEGFA gene expression levels positively correlate with VEGFA plasma serum levels and GA. Plasma VEGFA and monocyte gene expression of VEGFA was found to be significantly increased in samples obtained from term deliveries relative to samples obtained from preterm deliveries exposed to MVM (*n* = 24). Plasma VEGFA and monocyte expression of *VEGFA* was also significantly higher in samples exposed to inflammation compared with MVM. *P* < 0.002 using Bonferroni’s correction was used to determine significance for serum VEGFA concentration.

Four cytokines were found to be significantly different (*P* < 0.002 using Bonferroni’s correction for multiple comparisons) between Group A and Group B: IL1B, MCP1, TGFA, and VEGFA (Supplemental Tables 3 and 4; supplemental material available online with this article; https://doi.org/10.1172/jci.insight.155482DS1). From these 4 analytes, we then examined expression in our sample set for the correlating gene. We found that only *VEGFA* was differentially expressed in classical and intermediate monocytes (Figure 16). Term infants had significantly higher classical and intermediate monocyte *VEGFA* gene expression and plasma VEGFA concentration compared with preterm infants. Preterm infants with clinical chorioamnionitis and placental inflammation had higher classical and intermediate monocyte *VEGFA* gene expression and plasma VEGFA concentration than preterm infants with preeclampsia and MVM.

## Discussion

The predominant monocyte subsets present in our samples were classical (CD14^+^CD16^–^) monocytes, then intermediate (CD14^+^CD16^+^)monocytes, and finally nonclassical (CD14^–^CD16^+^) monocytes. Total monocyte composition did not correlate with GA or placental pathology. In terms of the dominant monocyte population isolated from blood, the compositions in our study populations differed from published literature, but variation between studies has been described in monocyte subpopulation composition in different models (29–32). The variation might be because of differences in patient populations and techniques used to quantify cord blood monocytes, which has often been highlighted as a limitation in comparing studies (33). Our study used 2 different techniques, monocyte enrichment and flow cytometry, to isolate monocyte populations with high congruency between both methods.

We found that variations in cord blood monocyte subpopulation composition correlate with placental histopathology. Specifically, samples with MVM and FVM on placental pathology had the highest percentage of nonclassical monocytes and a lower combined population of classical/intermediate monocyte when compared with monocytes exposed to inflammatory lesions or monocytes isolated from healthy pregnancies with normal placentas. Our finding is similar to peripheral blood monocytes isolated from mothers with preeclampsia, which is associated with MVM pathology. In the peripheral blood of preeclamptic mothers, the nonclassical monocyte (CD16^+^) population is increased, and CD16^–^ is decreased (30, 31, 34). However, the nonclassical monocyte population made up the smallest percentage of the total monocyte population and proved to be difficult to sequence. While alterations in monocyte populations exist, the functional differences between monocytes and the placental milieu’s influence on these differences are not well described. As a result, we chose to pursue transcriptomic profiling of the classical and intermediate monocyte populations.

Our data reveal that the primary contributors to global gene expression were GA and cell type. The biological processes that differentiate term from preterm monocytes in both classical and intermediate populations include genes involved in cell proliferation and cell adhesion. This included increased levels of heparin-binding epidermal growth factor-like growth factor (*HBEGF*) in monocytes isolated from term, healthy deliveries. In our data set, the most substantial decrease in gene expression of *HBEGF* occurred in monocytes isolated in the setting of MVM compared with the healthy group. These findings are important because activated monocytes secrete HBEGF, which functions in a paracrine manner to increase connective tissue differentiation, epithelial cell proliferation, and vascular smooth muscle division (35, 36). Recent literature has connected decreased *HBEGF* expression with histological chorioamnionitis, a hallmark of placental inflammation, while increased levels of HBEGF have been proposed to protect the intestinal epithelium in models of necrotizing enterocolitis, a disease attributed to prematurity (37–40). Our data suggest that global monocyte gene expression changes with GA and placental pathology, which may ultimately play a role in producing important mitogenic factors, suggesting that monocytes can be programmed before migrating to various tissues.

Overall, placental inflammation may induce chemotaxis, migration, and interaction with activated platelets in both monocyte subsets based on our transcriptomic data. GO analysis suggests that processes and functions related to leukocyte activation, monocyte maturation, and chemotaxis are activated in both monocyte subsets. Additionally, genes such as *CCR2*, *CXCR1*, *CXCR2*, and *CX3CR1* are upregulated in both monocyte subsets in the setting of placental inflammation. In sheep model models of chorioamnionitis, there is an increased amount of these chemotactic factors in fetal lungs after exposure to in utero LPS, while cord blood cytokine levels of the same molecules are increased with in utero inflammation (41–43). These same genes have also been linked to monocyte survival, differentiation into macrophages, and plaque accumulation in various inflammatory conditions such as atherosclerosis (44–46). Our data suggest that cord blood monocytes, much like peripheral blood monocytes, are modified in diseased states and are contributors to certain diseases.

Differential expression of endoglin-1 (*ENG1*) was unique to intermediate monocytes in our data set. Term intermediate monocytes upregulate *ENG1* compared with all 3 preterm groups based on WGCNA. ENG1 functions as a coreceptor for several ligands of the TGF-β family and is a recognized angiogenesis marker (47). The soluble form of endoglin, an antiangiogenic factor secreted by the placenta that impairs monocyte migration and differentiation into macrophages, increases in maternal plasma with preeclampsia associated with maternal vascular lesions (48–51). The association of increased *ENG1* expression with increasing GA is a potentially novel finding. Based on our results, intermediate monocytes are activated differently based on both GA and their interaction with the in utero environment.

When exposed to acute inflammatory lesions, intermediate monocytes upregulate neuregulin-1 (*NRG1*) compared with other groups in our data set, while classical monocytes downregulate *NRG1* when exposed to AI. NRG1 plays an essential role in lung development, surfactant production initiation, and continued lung inflammation (52, 53). Various studies have highlighted that in utero inflammation induces early lung maturity, that the process might be mediated by the macrophage/monocyte system, and that resulting inflammation may predispose neonates to bronchopulmonary dysplasia (BPD) (9–12, 54, 55). These data support epidemiologic findings that increased exposure to intrauterine inflammation leads to a decreased incidence of respiratory distress syndrome postnatally (7). Our study indicates potential sources for NRG1 production that may play a role in the early lung maturation observed in the setting of maternofetal inflammation.

By refining our data set to compare subjects with singular, well-defined placental pathology and the clinical feature associated with that placental pathology, we found further evidence to differentiate classical monocytes from intermediate monocytes in the preterm population. While many genes related to inflammation, including *BPI*, *LTF*, *MMP9*, and *MPO*, were upregulated in both subsets in the inflammation-exposed group, intermediate monocytes exposed to inflammation upregulated *TLR4* in our data set. This pattern recognition receptor leads to increased cytokine production in response to LPS (56). Increased *TLR4* expression has been described in maternal CD14^+^ monocytes and has been implicated in preterm delivery in the presence of inflammation (57, 58). Furthermore, TLR4 has been implicated in necrotizing enterocolitis and necrotizing enterocolitis–linked lung injury (59, 60). Here, we show that the fetal intermediate monocyte subpopulation might produce TLR4 following exposure to inflammation.

As research linking placental inflammation and monocyte function continues to emerge, data regarding the interaction between placental MVM lesions and monocyte subsets remains sparse. To our knowledge, our study is the first to describe the transcriptomic profile of fetal cord blood monocytes in the setting of MVM lesions and the clinical finding of preeclampsia. In our data set, classical and intermediate monocytes downregulate the expression of vascular endothelial growth factor A (*VEGFA*) in the setting of MVM lesions while plasma levels of VEGFA are higher in the inflammation and term groups. Our group has previously described how MVM lesions increase the likelihood of developing BPD and BPD-associated pulmonary hypertension, while also showing that exposure to MVM lesions decreases VEGFA (4, 61, 62). Our work reveals a potential source for VEGFA production, an important mitogen that may also play a role in the postnatal development of prematurity-related diseases.

Our data also reveal that MVM lesions with clinical preeclampsia are associated with differences in intermediate monocyte gene expression. In our data set, 642 genes were upregulated in this group, and GO enrichment analysis revealed that these genes were involved in IFN-γ signaling, MHCII receptor activity, and cell differentiation. The genes include multiple Human Leukocyte Antigens (*HLA*) genes *CD4*, *CCL3*, and *CSF1R*. This highlights a potential role for intermediate monocytes as antigen-presenting cells that interact with the adaptive immune system. In addition to these genes, the gene coding for the *MMP9* protein, a protein involved in extracellular matrix processing, was upregulated in this gene set. Interestingly, it has been shown that *MMP9*-KO mice have abnormal placenta and develop a preeclampsia phenotype (63). This dichotomy highlights the overall immune dysregulation that occurs in the setting of preeclampsia, with intermediate monocytes potentially playing a unique role in its pathophysiology.

While extremely limited by sample size, we gained insight into fetal nonclassical monocyte gene expression through our analysis. The majority of samples in the preterm group were female subjects with MVM on placental pathology and clinical preeclampsia. An expansion of the nonclassical monocyte subset occurs in the peripheral blood of pregnant mothers and fetal monocytes obtained from cord blood of growth-restricted infants with preeclamptic mothers (29, 30, 33, 64). An expansion of nonclassical monocytes may have contributed to the recovery of sufficient cells for RNA-Seq.

Our sequencing data suggest that nonclassical monocytes might play a role in the vascular pathology seen in preeclampsia. GO terms related to leukocyte adhesion, migration, and interactions with the vascular endothelium were enriched in the preterm group compared with the samples in the term group. The fractalkine receptor *CX3CR1*, linked to increased angiogenesis in diabetic placentas and early-onset severe preeclampsia, was upregulated in our samples exposed to placental MVM and preeclampsia (65, 66). In animal models, monocytes that upregulate *CX3CR1* preferentially differentiate into wound healing macrophages (67). While nonclassical monocytes that express *CX3CR1* patrol the vascular endothelium to remove debris and inflammatory damage (67–69). Our data provide insight into a specific source for fractalkine-mediated changes associated with preeclampsia and how they might affect the fetus. Although our data are provisional, we hope this information provides a framework and guidance for future studies that utilize more sensitive single-cell sequencing methods.

Our analysis also revealed that the method and timing of delivery impacted fetal monocyte gene expression. While this was not the purpose of our study, we did investigate the difference in monocyte gene expression based on vaginal delivery and cesarean delivery because epidemiologic data suggest that infants born via cesarean section carry a higher lifetime risk of autoimmune disorders and asthma (70–72). In our data set, we found classical and intermediate monocytes from term vaginal deliveries upregulated genes related to chemokine signaling, leukocyte activation, and leukocyte migration. The genes contained within these ontologies included *IL1B*, *MMP9*, and *VEGFA*. Our findings parallel those from other models that show leukocytes are activated prior to parturition and that various chemokines potentially play a role in the onset of parturition (22, 23, 73). While our data suggest a potential source for these chemokines, our data are limited, as we could not compare healthy term cesarean deliveries to term vaginal deliveries.

Our data also reveal that classical monocytes isolated from preterm vaginal deliveries upregulated T cell activation and type 1 IFN response genes. Our findings support recent literature suggesting preterm labor and chorioamnionitis activate T cells and implicate monocytes as a potential source for T cell activation (74–76). While data describing maternal and placental immune cell gene expression prior to delivery exists, our data suggest that specific fetal immune cell gene expression varies based on the timing of delivery and route of delivery. However, our sampling did not intend to compare monocyte gene expression based on the delivery method, and the distribution of pathology was not the same in the preterm vaginal delivery compared with the preterm cesarean delivery group.

This study had several other limitations. For example, we did not include term births with chorioamnionitis or preeclampsia, as our patient population was selected primarily to study the role of placental lesions and GA on monocyte subset biology. Furthermore, while preeclampsia occurs in pregnancies at preterm and term, we specifically set out to study how different conditions that lead to prematurity affect monocyte function as opposed to studying how preeclampsia affects monocyte function. Additionally, we focused specifically on the transcriptomic profile of monocytes, and as is the case with any transcriptome-based approach, the study provides broad information on what genes are expressed but not what genes are being translated into proteins. Since we were sampling at the time of delivery, we only have information at one time point. We attempted to overcome this limitation by sampling a sizeable GA range of infants, and we showed that GA is an important contributor to gene expression. Lastly, preeclampsia is a highly complex state that often reflects a chronic process, as the initial vascular dysregulation starts early in pregnancy. Further evaluation is needed to understand these processes and the integral role of fetal monocyte gene expression more comprehensively.

To our knowledge, this is the first study describing the transcriptomic profile of cord blood monocytes and their subsets in the setting of prematurity and exposure to various placental pathologies. Our data highlight the effect of GA and placental milieu on global monocyte gene expression. Our results highlight how there may be differences in monocyte subsets based on exposure to placental inflammation and how this potentially affects the 2 primary monocyte subtypes differently. This study’s findings may provide insight into the differences in prematurity-related outcomes and potential avenues for therapeutics that target specific pathways.

## Methods

### Patient enrollment.

Seventy mothers and their newborn infants were included in this study. Participants were prospectively enrolled through an ongoing biorepository in which all women delivering a liveborn infant at Prentice Women’s Hospital (Chicago, Illinois, USA) are eligible. Informed consent was obtained from all participants prior to participation. The 70 births were selected based upon their clinical profiles to include mothers with chorioamnionitis and/or preeclampsia, as well as a wide range of GAs. Infants with known congenital anomalies, infections, and genetic syndromes were excluded.

### Clinical data.

GA at birth was recorded as completed weeks and further categorized according to CDC classifications (77). Full-term was defined as > 37 completed weeks of gestation. Preeclampsia and other hypertensive disorders of pregnancy were defined according to the American College of Obstetricians and Gynecologists (ACOG) criteria (78). Chorioamnionitis was defined to occur when there was clinical evidence with histologic determination by placental pathology exam (27, 79). Placental pathology was obtained for all preterm deliveries when possible, and pathology reports were provided by the Department of Pathology at Northwestern Medicine (Chicago, Illinois, USA). Our pathology lab documents all pathologic placental features based on the Amsterdam Criteria, and all of the available features were tabulated (Supplemental Table 1) (80).

### Cord blood collection.

Delivery staff collected umbilical venous cord blood at birth into a cord blood bag or EDTA tube as previously described (62, 81). We exclusively collected umbilical venous blood for this study to help standardize collection protocol for our delivery team, to reduce the introduction of confounders or variations based on the source of collection (umbilical versus arterial), and to avoid interference with the collection of clinical samples. We stored cord blood specimens at 4°C and performed monocyte isolation within 36 hours of delivery. Corresponding cord blood plasma from all patients was aliquoted and stored at –80°C until assay.

### Monocyte isolation and enrichment.

Cord blood specimens were centrifuged at 240 *g*, 25°C for 10 minutes, and plasma and anticoagulant were removed. RBCs were lysed for 15 minutes at room temperature in the dark, using BD Pharm Lyse (BD Biosciences) and 10 mL lysis buffer per 1 mL cord blood. PBS was added to the tubes and centrifuged at 240 *g*, 25°C for 5 minutes. After removing the lysis buffer solution, the cells were washed with 2% BSA in PBS to remove residual lysis buffer. The cells were counted to obtain cell concentration and viability (Bio-Rad). A total of *n* = 36 samples were enriched using a pan-monocyte enrichment kit according to manufacturer’s protocol (Miltenyi Biotec). The enriched monocytes were then depleted of CD34^+^ stem cells utilizing a CD34^+^ isolation kit. The monocyte and CD34^+^ fractions were frozen in CTS Synth-a-Freeze (Gibco, Thermo Fisher Scientific) to –80°C and stored until use. The remaining samples (*n* = 34) underwent FACS to quantify immune cell composition. There were no differences in monocyte composition or monocyte subset composition when comparing samples that underwent only FACS and those that underwent monocyte enrichment and then FACS.

### Flow cytometry and FACS.

Flow cytometry and FACS were conducted at the Robert H. Lurie Comprehensive Cancer Center Flow Cytometry Core Facility at Northwestern University and were performed on a BD FACSAria Special Order Research Product (SORP) system. Cord blood monocyte subsets were identified based on expression of cell surface markers listed in Supplemental Table 2. Classical, intermediate, nonclassical, and CD34^+^ cells were collected. Cells were immediately lysed in RLT Plus Lysis Buffer following FACS and stored at –80°C until RNA isolation.

### RNA extraction.

Nucleic acid was extracted from thawed monocytes using the Qiagen AllPrep DNA/RNA Kits (Qiagen). The manufacturer instructions were used to separate RNA from DNA. All 70 patients had classical, intermediate, and nonclassical monocytes RNA extracted (*n* = 210).

### RNA-Seq.

The above RNA samples were processed through the RNA-Seq core in the Northwestern Division of Pulmonary and Critical Care Medicine (Chicago, Illinois, USA). Purified RNA was assessed for quality and quantity using the Agilent 4200 TapeStation with standard reagents (Agilent Technologies). CDNA preparation and ribosomal RNA depletion were performed using the SMARTer Stranded Total RNA-Seq Kit v2 - Pico Input Mammalian (Takara). Sequencing libraries were prepared using this NextSeq 500/550 High Output v2 kit (Illumina). Libraries were sequenced on an Illumina NextSeq 500 instrument with a target read depth of approximate 4 million to 8 million aligned reads per sample. We attempted to extract and sequence RNA from all 3 monocyte subsets. However, the nonclassical monocytes samples had poor sequence quality, and we found that an initial cell count of 2000 cells with extracted RNA quantity of at least 100 pg/μL was required for successful sequencing output and alignment.

The data discussed in this publication have been deposited in NCBI’s Gene Expression Omnibus and are accessible through GEO Series accession no. GSE195727 (https://www.ncbi.nlm.nih.gov/geo/query/acc.cgi?acc= GSE195727).

### Sequence alignment and downstream analysis.

Sequencing output was processed using Ceto (82). Sequences were aligned to hg38 using the STAR aligner. Aligned data were normalized and transformed using EdgeR. The IDEP 0.93 code was used in R (version 4.03) to further processes data (83). Differentially expressed genes were assessed with DeSeq2 using FDR cutoffs of 0.05 and 2-fold change (log_2_FC = 1). Output files were further analyzed and visualized in the Morpheus browser (84). Hierarchical clustering of samples and k-means clustering of genes using 1000 iterations was performed within Morpheus. Additionally, WGCNA analysis was performed using the CEMiTool (85). Filtered counts tables were normalized using variance stabilizing transformation, and FDR < 0.05 were again considered significant. Gene list output from k-means clustering the WGCNA was then used as input for GOrilla to search for significant GO terms for biological processes, function, and components (86, 87). FDR < 0.05 were considered significant.

### Multiplex immunoassays.

Simultaneous measurement of 30 analytes was performed by sandwich immunoassays using Luminex xMAP platform in magnetic bead format. The multiplexed assay beads were obtained from a commercially available kit, and the analytes are listed in Supplemental Table 3 (MilliporeSigma). Plasma samples were thawed on ice and prepared in 1:1 dilution and analyzed according to manufacturer’s instructions. All samples were run in duplicate with standard curves for each marker and controls on each plate.

### Statistics.

Patient demographics, clinical characteristics, biomarker levels, and placental data were compared using 2-way ANOVA or Kruskal Wallis for continuous variables and χ^2^ or Fisher’s exact tests for categorical data. *P* < 0.05 was considered significant for single comparison. Multiplex data were reported as median and IQR and analyzed using a Wilcoxon’s rank-sum test, with Bonferroni’s adjustment for multiple comparisons. Data analysis was performed using STATA/IC version 13.0 (StataCorp). Graphs were prepared using GraphPad Prism 8.0.

### Study approval.

The study was approved by the IRB of Northwestern University (IRB no. 00201858). Seventy mothers and their newborn infants were included in this study, and informed consent was obtained.

## Author contributions

AMS and RB are co–first authors on this manuscript. Both helped with designing the analysis, processing samples, collecting data, analyzing data, and writing this manuscript. AMS, KKM, RB, ETB, SS, and AVM designed the analysis; RB, AMS, KKM, and ETL collected data; AVM, ETL, HAV, ETB, LME, SS, and WAG contributed analysis tools; AMS, KKM, RB, ETB, and AVM performed the analysis; and AMS, KKM, and RB wrote the paper.

## Supplementary Material

Supplemental data

Supplemental table 2

Supplemental tables 3-4

## Figures and Tables

**Table 1:**
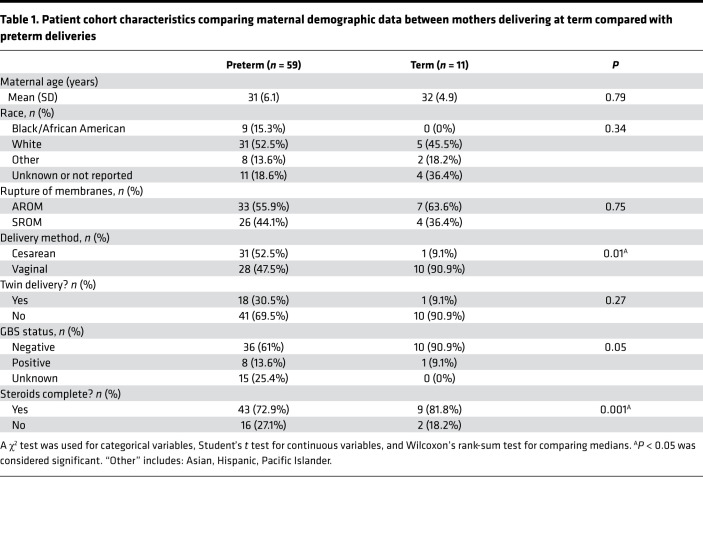
Patient cohort characteristics comparing maternal demographic data between mothers delivering at term compared with preterm deliveries

**Table 2:**
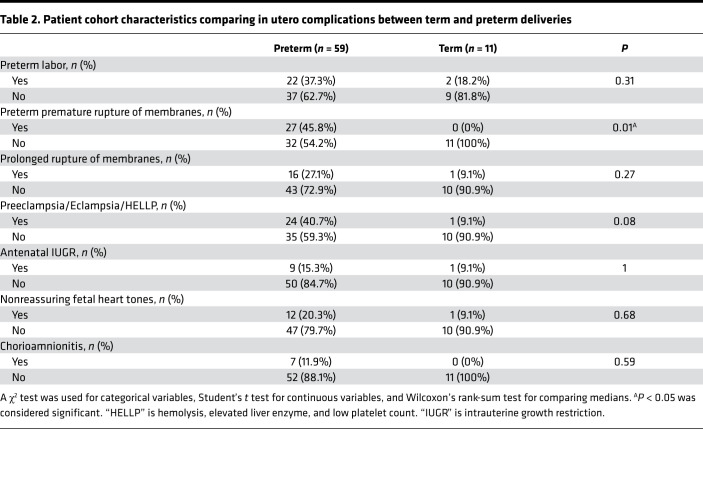
Patient cohort characteristics comparing in utero complications between term and preterm deliveries

**Table 3:**
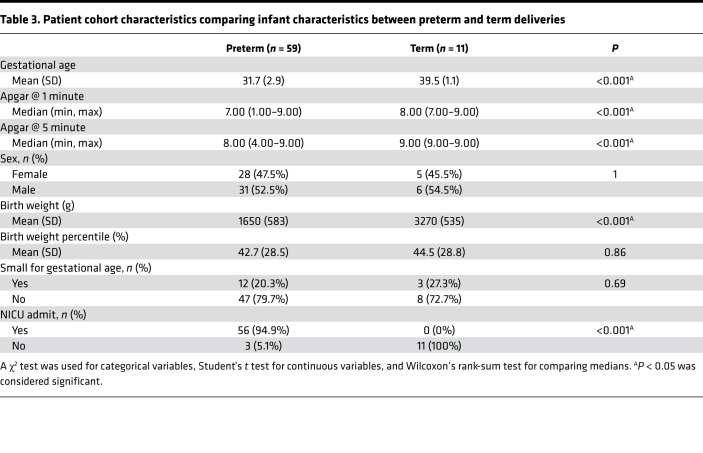
Patient cohort characteristics comparing infant characteristics between preterm and term deliveries

**Table 4:**
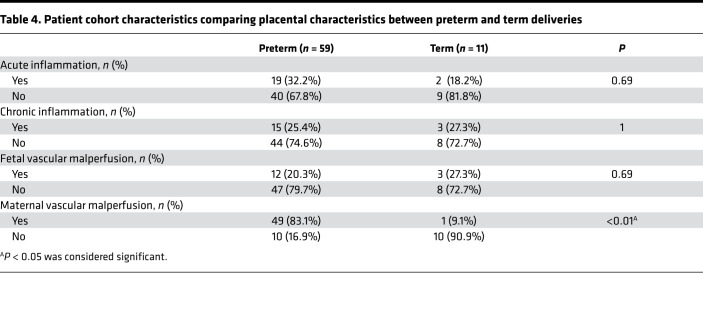
Patient cohort characteristics comparing placental characteristics between preterm and term deliveries

## Version 1 04/26/2022

In-Press Preview

## Version 2 06/08/2022

Electronic publication

## References

[1] Mir IN (2020). Impact of multiple placental pathologies on neonatal death, bronchopulmonary dysplasia, and neurodevelopmental impairment in preterm infants. Pediatr Res.

[2] Chisholm KM (2016). Correlation of preterm infant illness severity with placental histology. Placenta.

[3] Roescher AM (2014). Placental pathology, perinatal death, neonatal outcome, and neurological development: a systematic review. PLoS One.

[4] Mestan KK (2014). Placental pathologic changes of maternal vascular underperfusion in bronchopulmonary dysplasia and pulmonary hypertension. Placenta.

[5] Gagliardi L (2014). Association of maternal hypertension and chorioamnionitis with preterm outcomes. Pediatrics.

[6] Hartling L (2012). Chorioamnionitis as a risk factor for bronchopulmonary dysplasia: a systematic review and meta-analysis. Arch Dis Child Fetal Neonatal Ed.

[7] Lahra MM (2009). Intrauterine inflammation, neonatal sepsis, and chronic lung disease: a 13-year hospital cohort study. Pediatrics.

[8] Harris LK (2019). Placental bed research: II. Functional and immunological investigations of the placental bed. Am J Obstet Gynecol.

[9] Kramer BW (2007). Endotoxin-induced maturation of monocytes in preterm fetal sheep lung. Am J Physiol Lung Cell Mol Physiol.

[10] Kramer BW (2009). Prenatal inflammation and lung development. Semin Fetal Neonal Med.

[11] Kramer BW (2003). Monocyte function in preterm, term, and adult sheep. Pediatr Res.

[12] Kramer BW (2005). Endotoxin-induced chorioamnionitis modulates innate immunity of monocytes in preterm sheep. Am J Respir Crit Care Med.

[13] Passlick B, et al Identification and characterization of a novel monocyte subpopulation in human peripheral blood (1989). Blood.

[14] Krow-Lucal ER (2014). Distinct functional programming of human fetal and adult monocytes. Blood.

[15] Kapellos TS (2019). Human monocyte subsets and phenotypes in major chronic inflammatory diseases. Front Immunol.

[16] Medeiros LTL (2014). Monocytes from pregnant women with pre-eclampsia are polarized to a M1 phenotype. Am J Reprod Immunol.

[17] Rogacev KS (2012). CD14++CD16+ monocytes independently predict cardiovascular events: a cohort study of 951 patients referred for elective coronary angiography. J Am Coll Cardiol.

[18] SahBandar IN (2020). Relationship between circulating inflammatory monocytes and cardiovascular disease measures of carotid intimal thickness. J Atheroscler Thromb.

[19] Romero R (2006). Inflammation in preterm and term labour and delivery. Semin Fetal Neonatal Med.

[20] Romero R (2006). The preterm parturition syndrome. BJOG.

[21] Zhang Y-H (2017). Modulators of the balance between M1 and M2 macrophages during pregnancy. Front Immunol.

[22] Unal ER (2011). Maternal inflammation in spontaneous term labor. Am J Obstet Gynecol.

[23] Farine T (2017). Peripheral maternal leukocytes are activated in response to cytokines secreted by uterine tissues of pregnant women. Cell Mol Immunol.

[24] Goldstein JA (2020). Maternal-fetal inflammation in the placenta and the developmental origins of health and disease. Front Immunol.

[25] Ernst LM (2018). Maternal vascular malperfusion of the placental bed. APMIS.

[26] Kim CJ (2015). Chronic inflammation of the placenta: definition, classification, pathogenesis, and clinical significance. Am J Obstet Gynecol.

[27] Kim CJ (2015). Acute chorioamnionitis and funisitis: definition, pathologic features, and clinical significance. Am J Obstet Gynecol.

[28] Romero R (2018). The frequency and type of placental histologic lesions in term pregnancies with normal outcome. J Perinat Med.

[29] Alahakoon TI (2019). Characterization of fetal monocytes in preeclampsia and fetal growth restriction. J Perinat Med.

[30] Melgert BN (2012). Pregnancy and preeclampsia affect monocyte subsets in humans and rats. PLoS One.

[31] Groen B (2015). Immunological adaptations to pregnancy in women with type 1 diabetes. Sci Rep.

[32] Al-Ofi E (2012). Monocyte subpopulations from pre-eclamptic patients are abnormally skewed and exhibit exaggerated responses to toll-like receptor ligands. PLoS One.

[33] Faas MM, de Vos P (2017). Maternal monocytes in pregnancy and preeclampsia in humans and in rats. J Reprod Immunol.

[34] Vishnyakova P (2019). Role of the monocyte-macrophage system in normal pregnancy and preeclampsia. Int J Mol Sci.

[35] Mograbi B (1997). Human monocytes express amphiregulin and heregulin growth factors upon activation. Eur Cytokine Netw.

[36] Pan Z (1995). Platelet-activating factor stimulates transcription of the heparin-binding epidermal growth factor-like growth factor in monocytes. Correlation with an increased kappa B binding activity. J Biol Chem.

[37] Bermick J (2019). Chorioamnionitis exposure remodels the unique histone modification landscape of neonatal monocytes and alters the expression of immune pathway genes. FEBS J.

[38] Gaurav T (2019). Synthesis of substituted 2H-benzo[e]indazole-9-carboxylate as a potent antihyperglycemic agent that may act through IRS-1, Akt and GSK-3β pathways. Medchemcomm.

[39] Wei J, Besner GE (2015). M1 to M2 macrophage polarization in heparin-binding epidermal growth factor-like growth factor therapy for necrotizing enterocolitis. J Surg Res.

[40] Feng J (2005). Heparin-binding EGF-like growth factor (HB-EGF) and necrotizing enterocolitis. Semin Pediatr Surg.

[41] Yellowhair TR (2019). CXCR2 blockade mitigates neural cell injury following preclinical chorioamnionitis. Front Physiol.

[42] Shah TA (2010). Pulmonary and systemic expression of monocyte chemotactic proteins in preterm sheep fetuses exposed to lipopolysaccharide-induced chorioamnionitis. Pediatr Res.

[43] Otsubo Y (2017). Association of cord blood chemokines and other biomarkers with neonatal complications following intrauterine inflammation. PloS One.

[44] Tacke F (2007). Monocyte subsets differentially employ CCR2, CCR5, and CX3CR1 to accumulate within atherosclerotic plaques. J Clin Invest.

[45] Panek CA (2015). Differential expression of the fractalkine chemokine receptor (CX3CR1) in human monocytes during differentiation. Cell Mol Immunol.

[46] Landsman L (2009). CX3CR1 is required for monocyte homeostasis and atherogenesis by promoting cell survival. Blood.

[47] Rossi E (2019). Endoglin as an adhesion molecule in mature and progenitor endothelial cells: a function beyond TGF-β. Front Med (Lausanne).

[48] Leaños-Miranda A (2019). Soluble endoglin as a marker for preeclampsia, its severity, and the occurrence of adverse outcomes. Hypertension.

[49] Schmella MJ (2019). Plasma concentrations of soluble endoglin in the maternal circulation are associated with maternal vascular malperfusion lesions in the placenta of women with preeclampsia. Placenta.

[50] Venkatesha S (2006). Soluble endoglin contributes to the pathogenesis of preeclampsia. Nat Med.

[51] Luttun A, Carmeliet P (2003). Soluble VEGF receptor Flt1: the elusive preeclampsia factor discovered?. J Clin Invest.

[52] Dammann CE (2003). Role of neuregulin-1 beta in the developing lung. Am J Respir Crit Care Med.

[53] Hoffmann I (2010). Neuregulin-1, the fetal edothelium ad brain damage in preterm newborns. Brain Behav Immun.

[54] Schmidt AF (2020). Prenatal inflammation enhances antenatal corticosteroid–induced fetal lung maturation. JCI Insight.

[55] Sahoo D (2020). Transcriptional profiling of lung macrophages identifies a predictive signature for inflammatory lung disease in preterm infants. Commun Biol.

[56] Vaure C, Liu Y (2014). A comparative review of toll-like receptor 4 expression and functionality in different animal species. Front Immunol.

[57] Pawelczyk E (2010). Spontaneous preterm labor is associated with an increase proinflammatory signal transducer TLR4 recepter on maternal blood monocytes. BMC Pregnancy Childbirth.

[58] Cappelletti M (2020). Maternal regulation of inflammatory cues is required for induction of preterm birth. JCI Insight.

[59] Jia H (2016). Pulmonary epithelial TLR4 activation leads to lung injury in neonatal necrotizing enterocolitis. J Immunol.

[60] Leaphart CL (2007). A critical role for TLR4 in the pathogenesis of necrotizing enterocolitis by modulating intestinal injury and repair. J Immunol.

[61] Yallapragada SG (2016). Placental villous vascularity is decreased in premature infants with bronchopulmonary dysplasia-associated pulmonary hypertension. Pediatr Dev Pathol.

[62] Mestan KK (2017). Cord blood biomarkers of placental maternal vascular underperfusion predict bronchopulmonary dysplasia-associated pulmonary hypertension. J Pediatr.

[63] Plaks V (2013). Matrix metalloproteinase-9 deficiency phenocopies features of preeclampsia and intrauterine growth restriction. Proc Natl Acad Sci U S A.

[64] Faas MM (2014). Monocytes and macrophages in pregnancy and pre-eclampsia. Front Immunol.

[65] Szukiewicz D (2013). Fractalkine (CX3CL1) and its receptor CX3CR1 may contribute to increased angiogenesis in diabetic placenta. Mediators Inflamm.

[66] Siwetz M (2015). Placental fractalkine is up-regulated in severe early-onset preeclampsia. Am J Pathol.

[67] Olingy CE (2017). Non-classical monocytes are biased progenitors of wound healing macrophages during soft tissue injury. Sci Rep.

[68] Thomas G (2015). Nonclassical patrolling monocyte function in the vasculature. Arterioscler Thromb Vasc Biol.

[69] Auffray C (2007). Monitoring of blood vessels and tissues by a population of monocytes with patrolling behavior. Science.

[70] Darabi B (2019). The association between caesarean section and childhood asthma: an updated systematic review and meta-analysis. Allergy Asthma Clin Immunol.

[71] Kristensen K, Henriksen L (2016). Cesarean section and disease associated with immune function. J Allergy Clin Immunol.

[72] Sevelsted A (2015). Cesarean section and chronic immune disorders. Pediatrics.

[73] Romero R (2018). A role for the inflammasome in spontaneous labor at term. Am J Reprod Immunol.

[74] Luciano AA (2011). Preterm labor and chorioamnionitis are associated with neonatal T cell activation. PLoS One.

[75] Gomez-Lopez N (2019). Fetal T cell activation in the amniotic cavity during preterm labor: a potential mechanism for a subset of idiopathic preterm birth. J Immunol.

[76] Rueda CM (2015). Effect of chorioamnionitis on regulatory T cells in moderate/late preterm neonates. Hum Immunol.

[77] Barfield WD (2018). Public health implications of very preterm birth. Clin Perinatol.

[78] Shapiro-Mendoza CK, Lackritz EM (2012). Epidemiology of late and moderate preterm birth. Semin Fetal Neonatal Med.

[79] Gibbs RS (1977). Diagnosis of intra-amniotic infection. Semin Perinatol.

[80] Khong TY (2016). Sampling and definitions of placental lesions: Amsterdam Placental Workshop Group consensus statement. Arch Pathol Lab Med.

[81] Chaudhury S (2019). Variations in umbilical cord hematopoietic and mesenchymal stem cells with bronchopulmonary dysplasia. Front Pediatr.

[82] https://github.com/ebartom/NGSbartom.

[83] Ge SX (2018). iDEP: an integrated web application for differential expression and pathway analysis of RNA-Seq data. BMC Bioinformatics.

[84] https://software.broadinstitute.org/morpheus.

[85] Russo PST (2018). CEMiTool: a Bioconductor package for performing comprehensive modular co-expression analyses. BMC Bioinformatics.

[86] Eden E (2007). Discovering motifs in ranked lists of DNA sequences. PLoS Comput Biol.

[87] Eden E (2009). GOrilla: a tool for discovery and visualization of enriched GO terms in ranked gene lists. BMC Bioinformatics.

