# Supplementary material for: Placental dysfunction influences fetal monocyte subpopulation gene expression in preterm birth
Source: JCI Insight. 2022 Jun 8;7(11):e155482. doi: 10.1172/jci.insight.155482 (PMC9220934; doi:10.1172/jci.insight.155482)
Supplement: Supplemental table 2 [file jciinsight-7-155482-s104.pdf]

Supplemental Table 2: The following antibodies were used for FACS isolation of monocytes. Cord blood monocyte subsets were identified based on expression of CD45, CD14, CD16, CD15, CD3 CD20, CD56, HLA-DR, CD64 and CD34.

Dye	Antigen	Clone	Vendor	Cat #
V450	HLA-DR	L243	BD Biosciences	642285
BV510	CD45	HI30	BD Biosciences	563204
FITC	CD34	581	BD Biosciences	560942
PECF594	CD15	W6D3	BD Biosciences	562372
PECF594	CD3	UCHT1	BD Biosciences	562280
PECF594	CD20	2H7	BD Biosciences	562295
PECF594	CD56	B159	BD Biosciences	562289
PE-Cy7	CD64	10.1	BD Biosciences	561191
PE	CD14	MΦP9	BD Biosciences	562691
APC	CD16	3G8	BD Biosciences	561248
