# Supplementary material for: Placental dysfunction influences fetal monocyte subpopulation gene expression in preterm birth
Source: JCI Insight. 2022 Jun 8;7(11):e155482. doi: 10.1172/jci.insight.155482 (PMC9220934; doi:10.1172/jci.insight.155482)
Supplement: Supplemental tables 3-4 [file jciinsight-7-155482-s105.pdf]

Supplemental Table 3: Multiplex data comparing analytes plasma obtained from healthy placenta (Group 1), plasma obtained from samples exposed to inflammatory placental lesions (Group 2), and plasma obtained from samples exposed placental vascular lesions (Group 3). Samples with $p < 0.002$were considered significant based on Bonferoni Test.

	Group 1<br>N=6<br>Median [IQR]		Group 3<br>N=14<br>Median [IQR]	P
EGF	83.2 [79.2, 96.1]	18.6 [6.6, 62.2]*	13.4 [0, 51.3]***	0.006
Eotaxin	49.6 [40.0, 65.0]	59.4 [46.9, 66.8]	38.7 [26.9, 62.4]	0.526
FGF-2	85.5 [59.1, 167.2]	149.2 [99.4, 675.9]	44.4 [24.0, 107.8]	0.076
FLT-3L	38.2 [30.5, 41.5]	32.4 [27.1, 52.2]	25.1 [13.1, 33.8]	0.157
Fractalkine	129.9 [90.2, 132.1]	133.6 [125.4, 140.9]	95.6 [90.2, 139.2]	0.420
G-CSF	0.0 [0.0, 0.0]	5012.6 [3487.6, 10473.4]***	0.0 [0.0, 26.7]††	0.003
GM-CSF	0.0 [0.0, 0.0]	0.0 [0.0, 0.0]	0.0 [0.0, 0.0]	<0.001
IFN-g	0.0[0.0, 0.0]	5.5 [0.0, 15.5]**	0.0 [0.0, 0.0]†††	0.005
IL-1a	3.9 [2.6, 4.5]	26.1 [10.2, 94.9]*	2.0 [0.6, 4.2]†††	0.006
IL-1b	23.1 [17.6, 36.8]	21.5 [14.0, 305.6]	0.6 [0.0, 1.7]**,††	0.001
IL-1RA	353.4 [220.6, 540.0]	8570.8 [4055.3, 11518.7]*	70.9 [21.6, 1270.0]††	0.012
IL-2	0.37 [0.07, 0.61]	0.83 [0.49, 1.37]	0.13 [0, 0.25]*,††	0.005
IL-3	0.41 [0.23, 0.52]	0.56 [0.26, 0.83]	0.06 [0.04, 0.33]*,†	0.031
IL-4	1.7 [0.7, 2.9]	0.28 [0, 0.59]*	0.0 [0.0, 0.26]***	<0.001
IL-6	1.8 [1.3, 8.5]	324.1 [233.7, 10922.7]**	3.3 [0.5, 16.7]††	0.007
IL-8	5.5 [3.1, 58.9]	338.3 [160.5, 416.3]*	12.2 [3.8, 25.5]††	0.016
IL-10	6.1 [0.0, 8.3]	190.7 [20.2, 377.6]*	1.7 [0, 7.5]††	0.015
IL-12p40	232.9 [125.6, 373.3]	348.2 [262.8, 424.2]	181.8 [130.7, 255.0]	0.278
IL-12p70	3.9 [3.8, 4.2]	3.0 [2.5, 5.1]	2.1 [1.8, 3.5]	0.049
IL-13	24.0 [18.7, 31.8]	9.3 [6.2, 29.4]	5.0 [0.0, 17.6]**	0.020
IL-17a	0.50 [0.0, 3.0]	2.5 [0.0, 40.8]	0.0 [0.0, 1.1]	0.218
IP-10	41.3 [28.2, 52.0]	207.5 [82.0, 395.6]*	34.8 [27.4, 69.8]††	0.039
MCP-1	105.6 [90.1, 115.4]	1180.2 [633.2, 10524.2]***	532.4 [275.1, 734.6]**	<0.001
MCP-3	22.9 [20.4, 24.6]	20.9 [19.4, 23.5]	12.0 [6.7, 19.1]**,†	0.007
MIP-1a	22.0 [13.2, 29.0]	48.9 [39.4, 57.1]	11.3 [7.4, 18.6]*, †††	0.003
MIP1b	34.8 [32.2, 63.5]	56.2 [38.3, 77.1]	31.2 [22.6, 42.4]	0.117
TGF-a	8.9 [6.1, 10.1]	5.2 [3.8, 6.0]	1.9 [1.5, 2.4]***, †	<0.001
TNF-a	32.2 [27.1, 35.2]	79.1 [34.9, 152.9]	22.1 [14.9, 25.0]**, ††	0.002
TNF-b	2.6 [1.6, 3.2]	1.9 [0.0, 27.6]	0.0 [0.0, 1.2]**	0.021
VEGF-A	156.9 [111.8, 282.2]	43.2 [26.9, 58.5]	0.0 [0.0, 2.5]***, †	<0.001

*P<0.05, **P<0.01, ***P<0.001 vs. group 1
†P<0.05, ††P<0.01, †††P<0.001 vs. group 2
Groups compared using Kruskal-Wallis with posthoc Dunntest

Supplemental Table 4: Plasma exposed to MVM (Group 3) had significantly lower levels of IL-1b, IL-4, TGF-a, and VEGF-a when compared to plasma obtained from samples exposed to inflammatory placental lesions (Group 2), and plasma obtained from term samples (Group 1). MCP-1 was significantly higher in plasma accompanying inflammatory placental lesions (Group 2). Plasma obtained from term samples (Group 1) had the highest levels of VEGF-a.

	Group 1<br>N=6<br>Median [IQR]	Group 2N=4<br>Median [IQR]	Group 3<br>N=14<br>Median [IQR]	P*
IL-1b	23.1 [17.6, 36.8]	21.5 [14.0, 305.6]	0.6 [0.0, 1.7]**,††	0.001
MCP-1	105.6 [90.1, 115.4]	1180.2 [633.2, 10524.2]	532.4 [275.1, 734.6]	<0.001
TGF-a	8.9 [6.1, 10.1]	5.2 [3.8, 6.0]	1.9 [1.5, 2.4]	<0.001
VEGF-A	156.9 [111.8, 282.2]	43.2 [26.9, 58.5]	0.0 [0.0, 2.5]	<0.001

*P<0.05, **P<0.01, ***P<0.001 vs. group 1
†P<0.05, ††P<0.01, †††P<0.001 vs. group 2
Groups compared using Kruskal-Wallis
Posthoc Dunntest p < 0.002 were significant using Boneferroni Test
